# General practitioners’ and medical students’ current knowledge and attitudes toward non-pharmacological interventions for dementia

**DOI:** 10.3389/fmed.2025.1573251

**Published:** 2025-07-23

**Authors:** Lou L. Frankenstein, Lea Pickard, Philipp Franikowski, Georg Jahn

**Affiliations:** ^1^Department of Psychology, Chemnitz University of Technology, Chemnitz, Germany; ^2^Center for Psychotherapy, Humboldt-Universität zu Berlin, Berlin, Germany; ^3^Institute for Educational Quality Improvement, Humboldt-Universität zu Berlin, Berlin, Germany

**Keywords:** dementia, primary care, non-pharmacological interventions, occupational therapy, behavioral therapy

## Abstract

**Background:**

General practitioners in Germany infrequently prescribe effective non-pharmacological interventions for dementia patients. The aim of this study was to investigate general practitioners’ education, knowledge, and experiences as well as attitudes toward non-pharmacological interventions to identify potential strategies for increasing treatment quality.

**Methods:**

Medical students (*N* = 115) and practitioners (*N* = 19) responded to an online survey about the content of their medical studies regarding dementia and two non-pharmacological interventions, occupational therapy and behavioral therapy. Additionally, practitioners (*N* = 41) rated their assessment and usage of non-pharmacological interventions compared to pharmacological therapy for individuals with dementia. In-depth, semi-structured interviews were conducted with general practitioners (*N* = 12) to determine the context factors, beliefs, knowledge, and attitudes influencing prescription decisions.

**Results:**

Non-pharmacological interventions seem to be highly underrepresented in medical education. Pharmacological therapy is reported to be used more often, despite possible negative side effects and despite the proven effectiveness of non-pharmacological treatment. The general practitioners’ attitudes toward behavioral and occupational therapy were heterogeneous, but uncertainty was prevalent regarding budget regulations, and reservations to allocate resources to individuals with dementia became apparent.

**Conclusion:**

To help more people with dementia and their caregivers benefit from the positive effects of non-pharmacological interventions, general practitioners need to be better informed about these treatment options.

## Highlights

- Behavioral therapy helps people with dementia and caregivers adapt to the cognitive, physical, and behavioral changes and occupational therapy, which can be prescribed extrabudgetarily in Germany, helps to manage activities of daily living.
- Practitioners expect non-pharmacological interventions to be more effective than medication for the treatment of MCI and mild dementia.
- Medical students seem to learn little about occupational therapy and barely anything about occupational and behavioral therapy for people with dementia, and general practitioners report prescribing those therapies rarely and were largely unsure about budgeting regulations.
- Medical students and general practitioners need better knowledge of non-pharmacological interventions and practitioners wish for training on non-pharmacological interventions.

## Introduction

Worldwide, an estimated 55 million people live with dementia, and this number is expected to increase yearly (1). Most forms of dementia cannot be stopped or reversed by medical treatment. However, non-pharmacological interventions are promising for sustaining autonomy longer, helping people with dementia master their everyday life, and decreasing caregiver burden. Non-pharmacological interventions can support individuals in adapting to impairments, increasing the level of activity, slowing the progression of dementia, reducing secondary symptoms, and improving quality of life for people with dementia and their caregivers (2).

Based on growing evidence about the positive outcomes of non-pharmacological interventions such as occupational and behavioral therapy, the S3 guidelines for the treatment of dementia in Germany recommend these interventions (3). Dementia is disabling by definition and can cause secondary psychological symptoms such as depression, anxiety, and aggression, especially due to frustration and low self-efficacy resulting from difficulties pursuing everyday life and rewarding activities. In a recent article by Kamoga et al. 175 Ugandan caregivers were interviewed about the people with dementia they cared for and in 99% of cases one or more behavioral and psychological symptoms of dementia (BPSD) occurred, for example, depression (81%), hallucinations (75%), or anxiety (67%) (4). As defined by the WHO, healthy aging requires the maintenance of functional ability, which can be described by a set of parameters including cognition, locomotion, vitality, mental health, and sensory aspects that form the “intrinsic capacity” (5). Non-pharmacological interventions can contribute to reestablishing success in activities of daily living, foster rewarding activities, and counteract functional loss, consistent with the rationale of a function-focused care approach. They can support people with dementia and their caregivers in coping with changes in their everyday life and challenging behavior due to dementia (6). Sanchez-Valdéon et al. found that suspending a regular non-pharmacological intervention due to the COVID-19 pandemic lead to a 0.4-point decrease in Mini Mental Status Examination (MMSE) score per month compared to a 0.1-point monthly decrease measured before (delivered 5 days per week on a regular basis, comprising, e.g., cognitive stimulation, reality orientation, and gerontogymnastics). This indicates that patients substantially benefit from the non-pharmacological intervention (7).

Occupational therapy aims at regaining or maintaining independence as long as possible. The main components of occupational therapy are the structured analysis and practice of personally relevant aspects of everyday life [see, e.g., manuals (8, 9)]. This also includes adaptations of the living environment and the use of aids to facilitate activity and prevent falls. Additionally, it comprises education about dementia and advice regarding available resources for support. Gitlin et al. reported a reduced decline in dependency on help with instrumental activities of daily living in 93 families (control *N* = 78) after five 90-min sessions of occupational therapy (*d* = 0.42) (10). Graff et al. delivered ten 60-min sessions of occupational therapy to 68 individuals with dementia and their caregivers and observed increased daily functioning (*d* = 1.33) and a higher caregivers’ sense of competence (*d* = 1.20) (11). In the intervention group, 84% of individuals improved in terms of process outcome compared to 9% in the control group (11). In their meta-analysis of 15 trials, Bennett et al. reported an increase in activities of daily living (SMD = 0.61), a decrease in BPSD (SMD = −0.32), and an increase in quality of life in people with dementia (SMD = 0.76). Moreover, a small improvement in carer distress (SMD = −0.23) and an improvement in carer quality of life were found (SMD = 0.99). However, in five out of six studies that measured carer depression, there was no significant reduction found (12). As a recent study by Wenborn et al. showed, further evidence on the modes of effect is required. In their randomized controlled trial, 249 pairs of a person with mild to moderate dementia and a family caregiver received 10 h of Community Occupational Therapy in Dementia (COTiD-UK) and were compared to 219 pairs receiving treatment as usual. They found that 91% of the activity-based goals were achieved, but they did not find considerable benefits regarding activities of daily living (Bristol Activities of Daily Living Scale, BADLS; *d* = 0.12), quality of life (Dementia Quality of Life Measure, DEMQOL; *d* = 0.10), or depression in caregivers (Hospital Anxiety and Depression Scale, HADS-depression; *d* = 0.03) (13). COTiD was also implemented in Italy (COTiD-IT), and Lanzoni et al. analyzed barriers and facilitators during its implementation in a qualitative survey. The authors also identified health professionals’ lack of knowledge of occupational therapy as a major barrier (14). Guzzon et al. reported in their review on the cost-effectiveness of non-pharmacological interventions that occupational therapy can be highly cost-effective (15): Community Occupational Therapy versus usual care saved 1,748 € (“difference in mean total care costs per successful treatment”) (16).

Behavioral therapy changes observable behavior, cognition, emotion, motivation, and physiology. Along a disorder model and using targeted interventions the behavior can be reflected and modified (17). It aims to reduce possible BPSD, such as depression and anxiety. It comprises different elements, such as structuring everyday life to increase the level of activity and to establish routines, building pleasant activities and biographical work, as described in the neurological-behavioral therapy manual for people with mild dementia by Werheid and Thone-Otto (18). As part of a German dementia project the manual was evaluated in 100 participants with mild dementia (another 101 participants formed the control-group) and was rated helpful or very helpful by most of the patients, caregivers, and therapists. Kurz et al. found no effect on quality of life, behavioral disorders, and treatment satisfaction, but a non-significant effect on coping with everyday life and depressive symptoms (19). In the CBTAC (Cognitive behavioral treatment for patients with mild Alzheimer’s disease and their caregivers) modules are furthermore cognitive restructuring to modify the perception of dementia and concomitant life changes, life review to reactivate, reflect on, and integrate autobiographical memories, and behavior management for caregivers or couples counseling (20). In an evaluation by Forstmeier et al., 41 patients and their caregivers received approximately 25 sessions of CBTAC. There, reduced clinician-rated depression at post-test (T1, *d* = 0.59), reduced clinician- and self-rated apathy (*d* = 1.01 and 0.58, respectively), increased quality of relationship (*d* = 0.71), and increased informant-rated quality of life (*d* = 0.09) were found (21). In a meta-analysis Tay et al. also conclude that cognitive behavioral therapy can reduce depression and anxiety in individuals with mild dementia (22). It should be noted that the severity of dementia was low (0.5 to 2.0 on CDR, >11 on MMSE), the meta-analysis only included 11 studies with a total of 116 participants, and this number was further reduced due to attrition. This implies, that further RCTs with larger sample sizes are needed (22). Additionally, in their meta-analysis, Pinquart and Forstmeier reported a reliable improvement in the cognitive performance of individuals with dementia due to reminiscence interventions in five studies at follow-up (MMSE score, *d* = 0.50) that was increased compared to the effect size at posttest (*d* = 0.27) (23). As Hopkinson et al. reported in their meta-analysis, in 12 studies including 995 dementia caregivers altogether, behavioral therapy was shown to reduce depressive symptoms (*d* = −0.34), and in nine studies with 626 caregivers altogether, it was proven to reduce stress (*d* = −0.36) (24). A meta-analysis by Jütten et al. on a sample of 60 studies additionally concluded that behavioral therapy improved caregivers’ quality of life (Hedges’ *g* = 0.36), reduced stress (*g* = −0.18) and burden (*g* = −0.20), and increased caregivers’ sense of competence (*g* = 0.31) (25).

In Germany, health insurance is mandatory and follows the principles of solidarity and self-governance. Regulations for the coverage of therapy costs are developed by the health care system itself, which is self-governed by practitioners, psychotherapists, hospitals, health insurance providers, and representatives of the insured. The government just sets the legal framework conditions (26). General practitioners decide on prescriptions within monthly budget limits. A prescription is the doctor’s order for a medical device or treatment. It differs from the referral to a medical colleague. Currently, blank prescriptions are discussed for some indications. Therefore, the practitioner puts down the diagnosis but leaves further treatment options to the therapist. A European comparison by Schmachtenberg et al. identified models of good care practice as well as care gaps regarding the care supply for people with dementia in 17 countries (27). For Germany, they listed a lack of inpatient care as well as a lack of “outpatient general practitioners, geriatric, psychotherapeutic, and rehabilitative care for people with dementia in rural areas” (27), which might also be influenced by negative attitudes.

The guidelines for occupational therapy state that occupational therapy can be prescribed extrabudgetarily for people with dementia above the age of 70 years as well as for those with early-onset dementia (28). In the case of financial review, these prescriptions are not at the expense of the practitioners’ budget. Of the 8.8 billion business volume of cures performed in Germany (occupational therapy, physiotherapy, speech therapy, and podiatry), 15% are spent on occupational therapy. Thirty percent of occupational services are prescribed by general practitioners, and 20% are prescribed by psychiatrists/medical psychotherapists/neurologists. Of all occupational therapies, only 2% are administered due to dementia. Conversely, only approximately 1.5% of individuals with dementia currently receive occupational therapy in Germany (29). To explore how to reduce barriers to prescription, a project in Germany cross-linked 24 general practices with 8 occupational practices, 23 support offers, and 9 psychosocial helplines. In a preliminary interview, all the practitioners were unsure about the existing non-pharmacological interventions and their benefits. However, during the follow-up, all interviewed practitioners were convinced of the benefits of non-pharmacological interventions, especially occupational therapy. Regarding training, most general practitioners reported that what they had learned about dementia was going to positively influence their further professional activities (30). Ayeno et al. found in their cross-sectional study that in 96 participants, including paid caregivers, nurses, occupational therapists, lifestyle and wellbeing workers, and physicians, non-pharmacological interventions (such as behavior management, reminiscence therapy, modification of activities of daily living, validation therapy, physical activity, but also others, e.g., music therapy or social contact interventions) were rated more useful than medication. Two physicians argued that non-pharmacological interventions are client-centered and that they cause no or fewer side effects (31).

The guidelines for insurance-covered psychotherapy in Germany do not include behavioral therapy for people with dementia without secondary symptoms. This is based on the deliberation that dementia cannot be healed or significantly improved in the long term (32). Kessler (33) concluded that the psychotherapeutic health supply for older adults in Germany is generally insufficient and noted that, especially in the case of dementia, psychosocial interventions are far from being exhausted before medication is used. Kessler therefore recommends reviewing the limitation that psychotherapy for people with dementia can be administered only for those who have another main diagnosis, such as depression or anxiety.

Two international high-quality guidelines (2018 CANADA and 2018 NICE) recommend non-pharmacological treatment for people with dementia with BPSD. For agitation or depression, the CANADA guideline recommends behavioral therapy (besides others, such as social interaction or sensory therapy) as well as an improvement of the patient’s environment (34).

In focus group discussions, occupational and behavioral therapists reported partially insufficient prescriptions by general practitioners, criticizing the small number of prescriptions as well as incorrectly completed letters of referral (6). To remove these barriers toward effective treatment, it is highly important that general practitioners get well informed about non-pharmacological treatment options. Schoenmakers et al. state that general practitioners’ theoretical knowledge is good, while their guideline awareness is not and that caregivers criticize practitioners’ poor communication skills: The treatment of dementia regarding the caregiver was described as “time-consuming and highly frustrating” (35). On a positive note, Turner et al. reported that the majority of the 127 general practitioners who were asked agreed or strongly agreed that “much can be done to improve the quality of life of people with dementia” (79%) and for their caregivers (85%) (36). However, no more than 45% of the practitioners knew about support groups in the area for people with dementia, and only 50% knew a support group for caregivers (36). Alexander and Fraser identified insufficient knowledge about diagnosing mental health conditions such as dementia and about non-pharmacological treatments as two major factors that lead to suboptimal provision of services to people with mental health conditions (37). In their meta-analysis of 11 studies, Jennings et al. identified barriers to successful management of BPSD reported by general practitioners, including a lack of knowledge, unclear pathways of care, and time constraints (38). Furthermore, Giezendanner et al. distributed a survey to 4,460 Swiss general practitioners, 882 (21%) of whom took part (39). Most general practitioners (85%) favored an early diagnosis of dementia to enable prevention, planning, and support. The stated barriers to early recognition of dementia were time constraints (*r* = 0.59), paperwork (*r* = 0.59), discomfort in disclosing the diagnosis (*r* = 0.54), concerns about burden or stigmatization of patients (*r* = 0.54), possible suicide risk (*r* = 0.53), lack of necessity of timely diagnosis for patients or families (*r* = 0.49), inadequate financial remuneration (*r* = 0.45), and the opinion that resources should be allocated to late-stage dementia (*r* = 0.45). They also found an impact of negative attitudes held by general practitioners on the quality of disease management (39). Wangler et al. also emphasized the important role of general practitioners in the claim of support services by people with dementia and their caregivers and realized that some practitioners only consider pharmacological treatment of dementia (40). According to the World Alzheimer Report 2021, 33% of clinicians name the “belief that nothing can be done so why bother” as a barrier for timely dementia diagnosis (41).

To summarize, there are effective opportunities to improve the quality of life of people with dementia and their family caregivers non-pharmacologically. One of them is occupational therapy, which can be prescribed extrabudgetarily in Germany. However, if practitioners do not know the benefit of these interventions and the budget regulations, this is a major barrier to ensuring that the health care supply is met for people with dementia and their caregivers in line with the guidelines. Therefore, identifying information gaps beginning in medical studies is sensible. Additionally, pathways for interdisciplinary exchange are necessary to enable communication between general practitioners and therapists.

Our aim was to exploratively investigate the following research questions:

What do medical students and practitioners in Germany learn and know about occupational and behavioral therapy for people with dementia? Is there a need for further information?

How do practitioners make decisions regarding their prescriptions, and what aspects have an impact on them? Do they favor pharmacological or non-pharmacological interventions?

And what are the requirements for successful interdisciplinary exchange?

## Materials and methods

### Mixed methods

**Figure 1:**
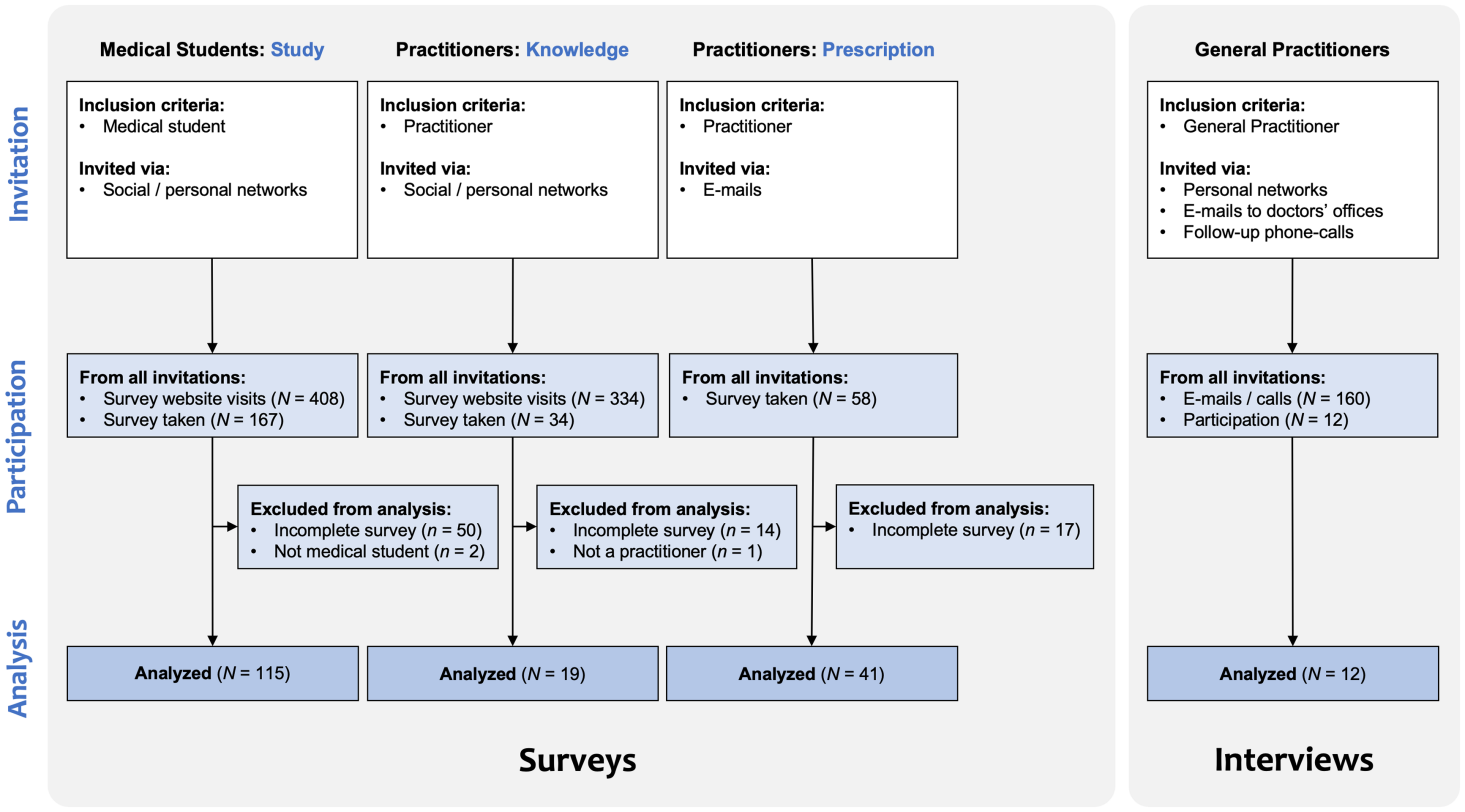
Recruitment procedure.

A mixed methods design was chosen that included surveys of medical students and practitioners as well as qualitative interviews with general practitioners to determine their education, knowledge, experiences, and attitudes toward non-pharmacological interventions for people with dementia and their caregivers (Figure 1). The instruments were discussed in a scientific colloquium and tested for time expenditure and comprehensibility by students. Medical students were included for insight into recent educational practices and practitioners, regardless of specialty, for common beliefs on non-pharmacological therapy approaches, also compared to pharmacological approaches. Additionally, general practitioners were interviewed about their knowledge and attitudes about people with dementia, their caregivers, treatment provision, and interdisciplinary exchange in general care.

Quantitative data were analyzed using R and RStudio (42), and qualitative data were analyzed using MaxQDA (43).

### Study survey

The first online survey was used to gain insight into the content of medical studies, especially regarding dementia, occupational, and behavioral therapy. The survey was administered using *SoSci Survey* (44). It addressed medical students and was distributed through different social media channels. The first author sent the link to acquaintances with relations to medical students and became a member of several Facebook groups for medical students and practitioners where the survey was then posted. The questions were created by the authors according to the research questions and included 6 closed- and 6 open-ended questions. The survey comprised demographic information, questions about the contents of the medical studies, associations, and knowledge regarding occupational and behavioral therapy, and miscellaneous information (the survey can be found in the Supplementary materials). The frequencies of the answers regarding the content of the medical studies were evaluated and compared by deriving effect size r from non-parametric Wilcoxon tests (45), separately for semester groups. The ratings of occupational and behavioral therapy on dimensions such as importance or pleasantness and the expected methods were averaged.

#### Participants

A total of 408 individuals accessed the link, 167 students voluntarily participated in the survey, and 117 completed it, two of these students were excluded because they were not medical students. Most of the participating students were from Berlin (*n* = 98), Mecklenburg-Western Pomerania (*n* = 24) and Bavaria (*n* = 23); altogether, nine of the 16 federal states were included. Eighteen students were in their first year, seven in the second, 11 in the third, 23 in the fourth, 29 in the fifth, 14 in the sixth, 12 in the seventh year, and one was above the 16th semester.

### Knowledge survey

A second online survey was designed for a closer look at practitioners’ knowledge of occupational and behavioral therapy. The survey was again administered using *SoSci Survey* (44). It addressed practitioners and was distributed through recommendations within the first authors’ personal network as well as in groups on Facebook. The questions were created by the authors according to the research questions and included 8 closed- and 3 open-ended questions. The survey comprised demographic information, questions about the contents of the medical studies, prescription practices, interdisciplinary exchange, and miscellaneous information (the survey can be found in the Supplementary material). Response frequencies regarding knowledge, prescription preferences, obstacles, and interdisciplinary exchange were determined.

#### Participants

A total of 334 people accessed the link, 34 took part in the survey, and 20 completed it, one of whom was a student and was therefore excluded from further analyses. Eleven of the 19 remaining participants identified as female and eight as male. Five were 20 to 29 years old, seven were 30 to 39 years old, six were 40 to 49 years old, and one was 50 to 59 years old. The participating practitioners had studied in 10 different federal states and were currently working in 10 different federal states, of those, who indicated their field of expertise, most were general practitioners, internists (*n* = 4 each), and psychiatrists (*n* = 3). The practitioners participated voluntarily after being informed about the purpose of the survey.

### Prescription survey

A third online survey was designed to determine whether and to what extent practitioners favor pharmacological or non-pharmacological therapy for treating dementia. The survey was again administered using *SoSci Survey* (44). It addressed practitioners and was distributed via email to 20 practitioners’ offices in each federal state of Germany, adding up to a total of 320 invitation emails. The questions were created by the authors according to the research questions and included 52 closed-ended questions and 13 open-ended questions. The survey was subdivided into demographic information, experience with pharmacological and non-pharmacological therapy, a comparison of pharmacological and non-pharmacological approaches, extrabudgetary prescription, barriers, and multimodal therapy (the survey can be found in the Supplementary material). Practitioners’ ratings of the effectiveness of non-pharmacological and pharmacological interventions for people with dementia were averaged and a within-subjects ANOVA with Greenhouse–Geisser correction was conducted to examine the effects of treatment, severity, and symptom category. Post-hoc contrasts were estimated to derive effect size *r* for the differences between pharmacological and non-pharmacological treatment (45). Regarding the questions on knowledge, frequencies were evaluated.

#### Participants

Fifty-eight doctors took part, and 41 completed the survey. Twenty-seven participants identified as male and 14 as female. Participants’ age ranged from 29 to 73 years (*M* = 50.6). The participants in the prescription survey were mainly general practitioners (*n* = 22), followed by neurologists (*n* = 11), and psychiatrists (*n* = 9). The participants had completed their medical studies between 1975 and 2020.

### Interviews with general practitioners

Twelve semi-structured interviews were conducted to obtain more detailed insights into general practitioners’ knowledge and attitudes toward dementia, occupational therapy, and behavioral therapy and about their experiences and wishes regarding interdisciplinary exchange. The interview questions can be found in the Supplementary material. Invitations were sent to acquaintances with relations to practitioners, resulting in four interviews. Additionally, 160 invitations were sent via e-mail to general practitioners from all federal states in Germany, selecting the first ten search results for general practitioners with a website and an e-mail address in each federal state. Besides automatic responses, none of the e-mails were answered. For this reason, the 160 doctors’ offices were then called, resulting in eight more interviews. The general practitioners participated voluntarily after being informed about the purpose of the survey. The interviews were scheduled for 30 min and lasted between 20 and 90 min. The survey comprised 34 basic questions covering demographic information (4 questions), occupational and behavioral therapy (4 each), diagnosis (3), people with dementia (3), relatives (3), recommendations (2), prescription practices (3), practitioner knowledge (2), information seeking (1), interdisciplinary exchange (2), future notices (1), and final questions (2). The interview format was preferred over a survey, because it was an opportunity to gain more detailed insight by asking follow-up-questions as needed. Most questions were open, so the answers were not biased by expectations. Due to the COVID-19 pandemic, all but two interviews were conducted via telephone. All interviews were recorded for the purpose of transcription, with consent provided prior to the recording. The recordings were then transcribed manually and deleted afterwards. The interviews were evaluated qualitatively. The answers were clustered and evaluated, for example in terms of frequencies. MAXQDA was used for the qualitative analysis (43).

#### Participants

The age of the 12 participants (seven female, five male) ranged from 27 to 65 years (*M* = 49.4 years). The participants were general practitioners, worked in nine different federal states of Germany and had studied in nine different federal states. One had studied abroad.

## Results

### Study survey

**Figure 2:**
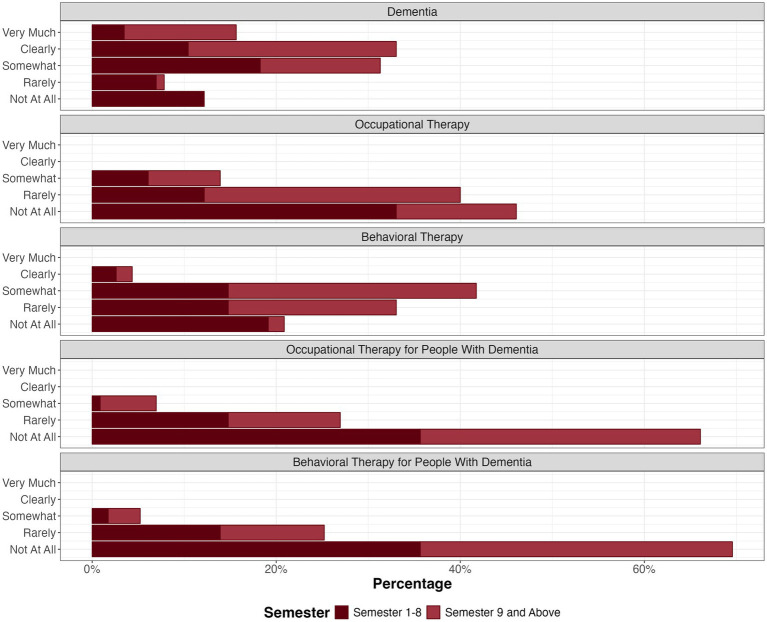
Focus on dementia, occupational therapy, and behavioral therapy in medical studies.

The students were asked to what extent dementia, occupational, and behavioral therapy were covered in their studies. The participants were differentiated by duration of study up to year four, including semesters up to eight (*n* = 59), and duration of study nine semesters and above (*n* = 56). Dementia has been discussed regularly, especially in late semesters (Figure 2), whereas not as much time seems to be spent on non-pharmacological therapy, particularly during semesters one to nine, with occupational therapy being less covered than behavioral therapy (*r* = 0.53 for semesters one to nine and *r* = 0.63 for the semesters above). Occupational and behavioral therapy, specifically for people with dementia, were not addressed much at all (*r* = 0.03 for semesters one to nine and *r* = 0.20 for the semesters above).

The same order of medical content was found for *Amboss*, a provider of medical content for medical students and practitioners: Amboss included 103 chapters on dementia, 52 chapters on behavioral therapy and 37 chapters on occupational therapy (46).

When asked for the first three associations related to behavioral and occupational therapy that come to their minds, most students answered “psychotherapy”/ “therapy” and associations connected to psychological or psychiatric symptoms (e.g., “fear,” “disturbance,” “depression,” “compulsion”), the specific focus of the therapy (“behavior,” “change”), or certain methods such as “confrontation” or “conditioning.” Interestingly, behavioral therapy has also been associated with “everyday life,” which at the same time was the most frequent association with occupational therapy. In addition to descriptions of the practical nature of occupational therapy (e.g., “help,” “movement,” “ability,” “practice”) and typical areas of application (such as “stroke”), there were also associations that reflect prejudices or one-sided experiences with this approach: “tinkering,” “pottery,” and “basket braiding.”

**Figure 3:**
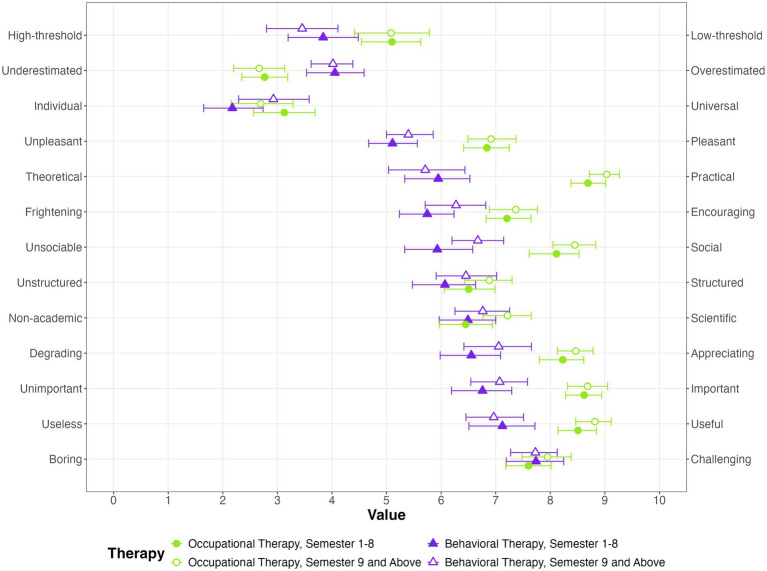
Occupational and behavioral therapy for dementia, rated by medical students.

The students were then asked how they would rate occupational and behavioral therapy on dimensions such as importance or pleasantness (Figure 3). Both approaches were rated as highly useful and rather challenging. Both were appraised to be underestimated by students in early semesters, occupational therapy was rated increasingly underestimated over the course of semesters. Occupational therapy was expected to be more useful, more practical, more social, and more important than behavioral therapy.

Figure 3 displays the scale means and their 95% confidence intervals.

**Figure 4:**
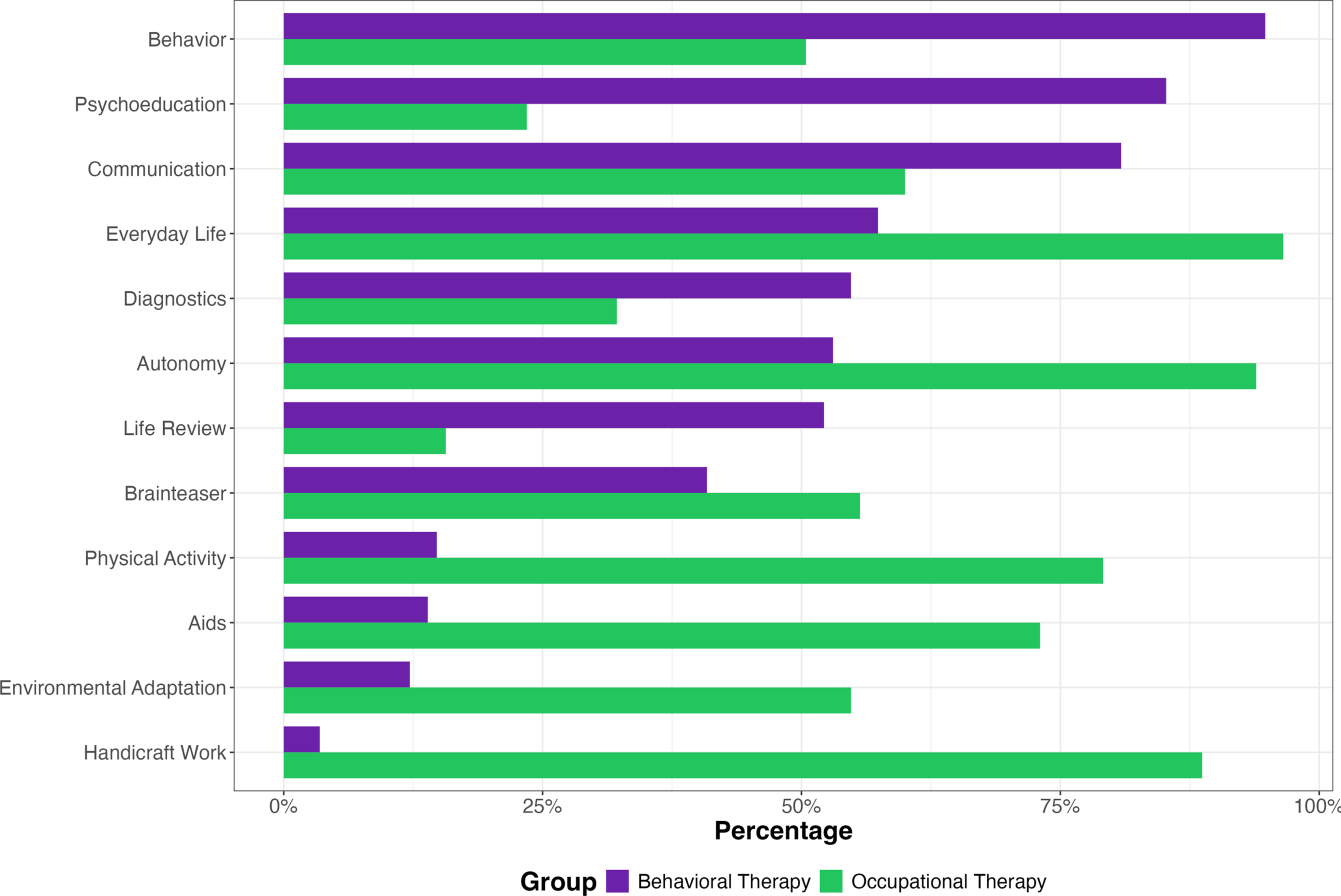
Mean ratings of the content of behavioral and occupational therapy expected by medical students.

When asked about the relevance of the selected components to both approaches, the medical students rated “behavior,” “psychoeducation,” and “communication” as the most central for behavioral therapy, followed by “everyday life.” For occupational therapy, they rated “everyday life,” “autonomy,” and “handicraft work” as the most important, followed by “physical activity” and “aids” (Figure 4).

### Knowledge survey

In the second online survey, the emphasis was still on education but regarding practicing doctors. The same pattern emerged as in the Study-Survey: Dementia was reported to be part of the medical studies, and behavioral therapy was also considered to some extent. Occupational therapy was less focused on, and behavioral or occupational therapy for people with dementia was rarely addressed or not at all (a figure displaying this can be found in the Supplementary material).

**Figure 5:**
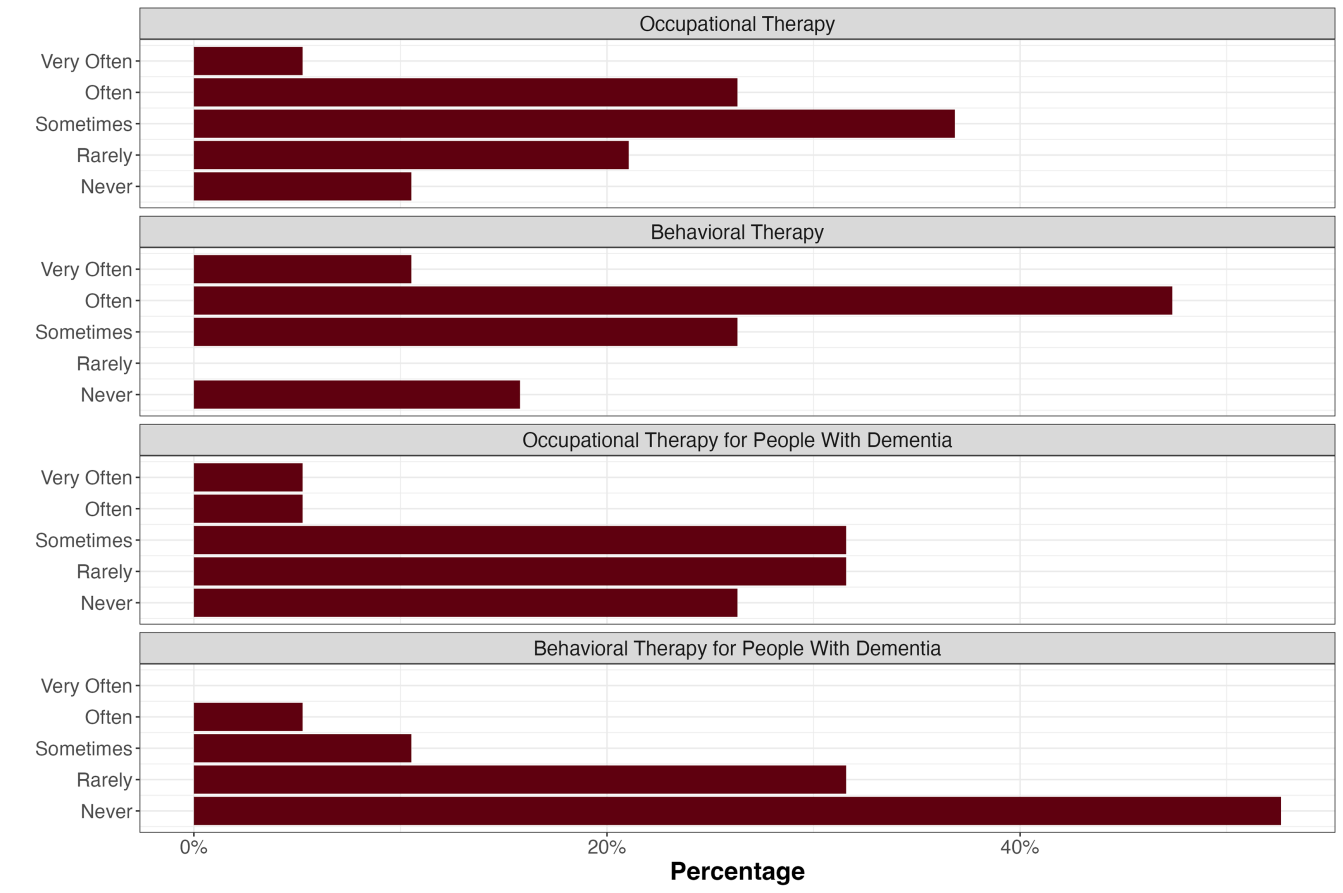
Practitioners’ prescription decisions regarding occupational and behavioral therapy.

Additionally, the practitioners were asked about their prescription decisions as well as their preferences regarding documentation and interdisciplinary exchange. As shown in Figure 5, behavioral therapy is prescribed more often than occupational therapy by the participating practitioners. A third of the practitioners reported having prescribed occupational therapy *never* or *rarely*, and three practitioners had never prescribed behavioral therapy. Two general practitioners reported to often or very often prescribe or recommend non-pharmacological approaches for individuals with dementia.

In an open query, the participants had the opportunity to name reasons for potential reluctance to prescribe non-pharmacological interventions for people with dementia and their caregivers. Here, the desire for more information or knowledge (*n* = 6), clear recommendations or evidence (*n* = 4), security (*n* = 3), more resources such as therapists or treatment places (*n* = 3), and a fear of financial regress claims (*n* = 3) were expressed. Six answers suggested that the prescription of occupational and behavioral therapy for people with dementia did not occur in the work context of the respective participants.

Regarding the information that practitioners expected from therapists, 16 practitioners would like to be informed about interim progress and would like to receive a recommendation for further prescription, 14 would expect a final report, 12 would like to be informed about psychological abnormalities, 10 about physical abnormalities, and one stated to welcome a summary for each session. One participant used the open query to express the desire for more information on non-pharmacological therapy options and their acceptance. When asked about their preferred channels for interdisciplinary communication, 18 practitioners preferred the written form, three would like to receive the information via telephone, and three would welcome a personal exchange. In an open query, one practitioner added “e-mail” as a good means; however, this is not in line with the current data protection guidelines.

Finally, the practitioners were invited to leave notes on further components that should be included in non-pharmacological therapy for people with dementia and their family caregivers. They listed the inclusion of exercise and coordination training, targeted nutrition, including micronutrient supply, memory training, and personal exchange on recent experiences.

### Prescription survey

**Figure 6:**
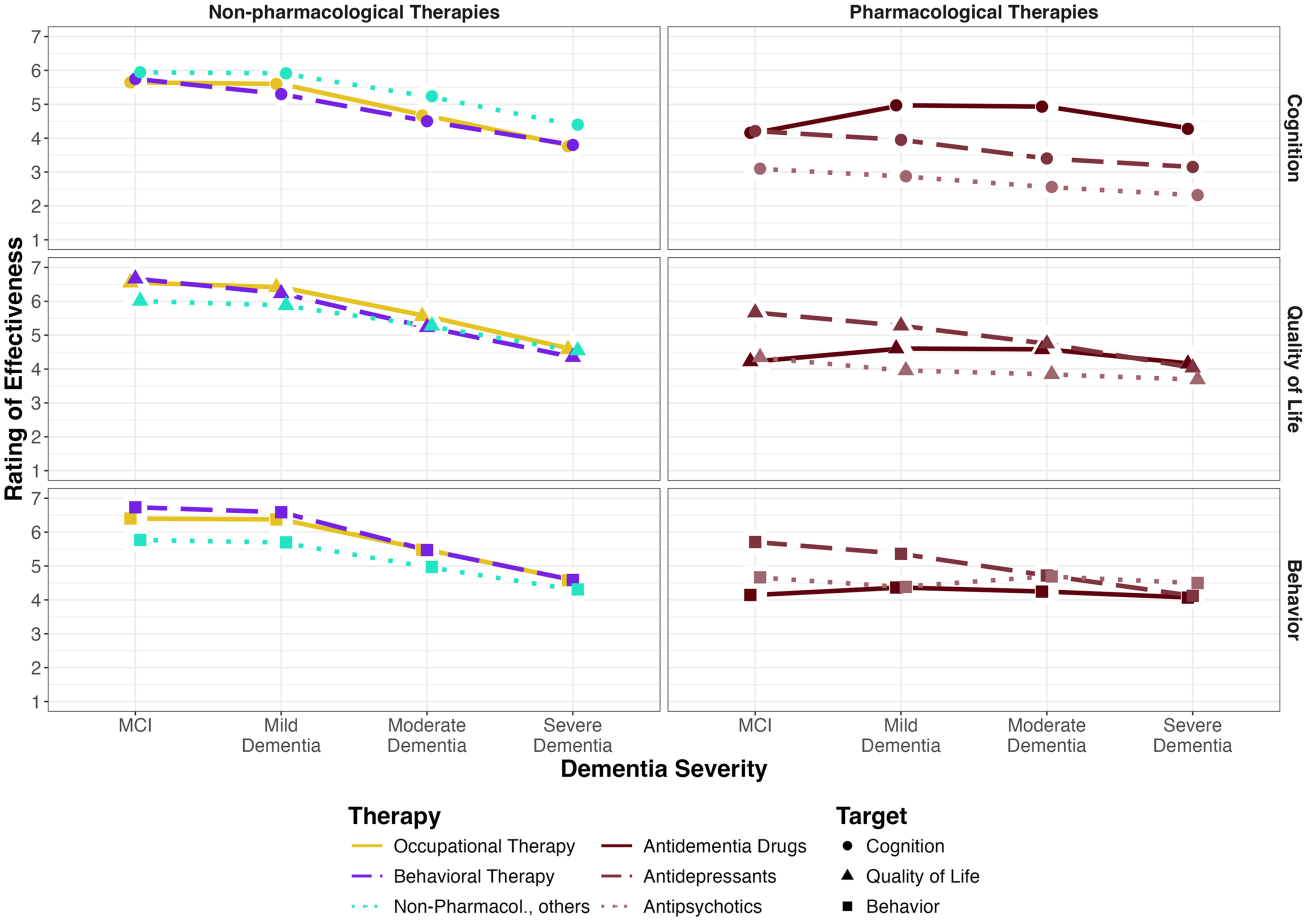
Mean rating of effectiveness of non-pharmacological and pharmacological interventions on cognitive performance, everyday functioning and quality of life and behavior.

A within-subjects ANOVA was conducted to examine practitioners ratings of the different treatments (pharmacological and non-pharmacological therapy) regarding their effectiveness on the symptom categories (cognitive performance, quality of life / functionality in everyday life and behavior) across the severity levels (MCI, mild, moderate, and severe dementia). Main effects of treatment, *F*(1, 26) = 35.21, generalized *η*^2^ = 0.18, severity of dementia, *F*(1.4, 37.3) = 35.86, *η*_g_^2^ = 0.18, and symptom category, *F*(1.7, 44.3) = 20.96, *η*_g_^2^ = 0.06 were found. There was a strong two-way interaction effect between treatment and severity, *F*(1.4, 36.0) = 13.04, *η*_g_^2^ = 0.07. Non-pharmacological therapy was rated as clearly superior to pharmacological therapy for MCI (*r* = 0.88), followed by mild dementia (*r* = 0.86), moderate dementia (*r* = 0.45), and severe dementia (*r* = 0.18). The magnitude of superiority in rated effectiveness decreases with increasing severity. Also, the expected effectiveness generally decreases with increasing severity (Figure 6). Non-pharmacological interventions were expected to be more effective than pharmacological interventions across all severity levels for each symptom category, except for the category behavior in severe dementia, where practitioners expected pharmacological and non-pharmacological treatment to be equally effective and the category cognitive performance, where practitioners expected antidementia drugs to be more effective in patients with moderate or severe dementia (Figure 6).

The practitioners were also asked which therapies they knew for the treatment of dementia. Among the medications, memantine was the most well-known drug (*n* = 31), followed by donepezil and galantamine (*n* = 29 each) and rivastigmin and antipsychotics (*n* = 26 each). Twenty-five stated to know antidepressants for dementia therapy. The best-known psychosocial intervention in dementia treatment was occupational therapy (known by 25 practitioners). Twenty practitioners knew that occupational therapy can be prescribed extrabudgetarily. The second most well-known therapy was memory therapy (*n* = 22), followed by art and music therapy, and cognitive stimulation therapy (*n* = 20 each). Behavioral therapy and physical activity were the least well-known approaches for people with dementia (*n* = 19 each).

### Interviews with general practitioners

The interviews were also held to investigate the general practitioners’ attitudes toward dementia, behavioral and occupational therapy, and intervention application. “People with dementia” was not further specified to get an impression of the severity and types of cases that doctors have in mind spontaneously. Obstacles to delivering non-pharmacological therapies were identified and discussed. The main findings are reported below.

#### People with dementia

The patients were described as rather inconspicuous (*n* = 3) and some as aggressive (*n* = 3). Typical peculiarities were misunderstandings and misremembering of information related to medication (*n* = 3) or appointments (*n* = 2). Another difficulty was the perceived decrease in understanding (3). One practitioner said he would respond by lowering his expectations. One major topic of conflict was driving cessation (*n* = 3). People with dementia were reported to be usually ashamed of the disease and often try to hide it (*n* = 8): “*Dementia is something that causes a lot of shame and helplessness.*”

The predominant wishes of the patients were uniformly to solely remain in the home environment (*n* = 5), to maintain autonomy (*n* = 3), and familiar surroundings with a stable social network (*n* = 3). At the same time, the main perceived concerns were to lose autonomy or fear of dependency (*n* = 5), fear of the future (*n* = 3), having to move to a nursing home (*n* = 2), having to leave one’s partner alone (*n* = 1), and fear of stigmatization (*n* = 1). However, there is also the fear of becoming a burden to the family (*n* = 2): “*What everyone wants is to remain autonomous. And what that means then, I think, is different, but, if possible, not in a nursing home, if possible, not to be a burden to anyone, if possible, to die independently.*”

Regarding the progression of dementia, a negative expectation was expressed: “*Because with dementia, very rarely truly, at least with the collective I have, I expect truly great success*,” “*from my point of view there is not much left to save*,” or even “*in the end it goes downhill anyway*” (*n* = 2), and “*there is nothing left then*.” One practitioner admitted: “*Then, you subconsciously invest in nondemented patients more.*” By a single practitioner, dementia was described as rather attractive in general practice in a commercial sense due to its chronic progression.

#### Diagnosis

For diagnosing dementia, the MMSE was most popular (*n* = 8), followed by the Clock Drawing Test (*n* = 7), and the DemTect (*n* = 6). Most practitioners (*n* = 9) reported being content with these tools. Criticism included the predominance of the personal impression over test results and the humiliating nature of some test procedures. Additionally, a lack of time was reported, which prevents practitioners from performing basic tests on a regular basis. Some participants felt that “*there are many opportunities for optimization for us to practice early notice*.” Another aspect mentioned by two practitioners was the will of the patients: “*There is also something like a right not to know.*”

#### Occupational therapy

Eleven practitioners reported regularly prescribing occupational therapy. Six of them also considered occupational therapy for people with dementia, four had not prescribed it yet (one of them was just about to work in general practice). Describing occupational therapy, practitioners named an improvement in everyday skills (*n* = 4), handicraft work (*n* = 3), and stroke (*n* = 2). Most of them seemed to value the approach (*n* = 7). Asked about the relevance of occupational therapy within their medical studies, one did not remember, and all the other participants said, it did not matter at all (*n* = 6) or it did not matter much (*n* = 5). The experiences with occupational therapy ranged from none or very few (*n* = 4) to experience during internship (*n* = 2), in hospital (*n* = 3), or in a geriatric center (*n* = 1). There were positive as well as negative statements about occupational therapy. Seven practitioners perceived occupational therapy as useful or important, for example, *“I think it is good, especially in geriatrics, when it comes to everyday practical things,”* and described it as “*ultimately a kind of life-help.”* Criticism toward occupational therapy included skepticism toward the effectiveness (*n* = 2) and the special suitability of this approach (*n* = 3): “*Everyday life itself – bringing the cup to the mouth or getting dressed – that’s enough of occupational therapy*.” A specific criticism brought forth referred to the extent of the therapy (*n* = 2), for example, phrased “*I’m always delighted when someone says: Enough now. There is no need for further therapy. Or it does not help. Or maybe a break might be good. In my opinion, that happens too rarely.*” Another treatment was preferred: “*You are more likely to be heading toward physiotherapy than occupational therapy*” or “*Coordinative and cognitive training, that is what it is about. But not about occupational therapy*.” Patients’ reactions to occupational therapy were generally positive, as 11 practitioners reported. Three practitioners said, however, that sometimes the patients did not know about occupational therapy in advance: “*Mostly they do not know what it is, but when they do it, they think it is good*.”

#### Blank prescriptions for occupational therapy

A blank prescription leaves the therapy procedure to the therapist. Seven practitioners thought positively about blank prescriptions, four of whom stressed the therapists’ expertise: “*The occupational therapist, he is the specialist in his area, and therefore, he can do what he thinks is necessary, right? That’s very ok.*” Five practitioners did not like the idea: “*I do not know at all that there is such a thing, and I will never use that.*” Two of them argued that they were still responsible: “*I am ambivalent about that. So, I would like to know what is being done. Because my name is underneath it.*” Another two were convinced that a practitioner knows better what to prescribe: “*I do believe that I know better what to prescribe for patients than an occupational therapist.*”

#### Behavioral therapy

One practitioner reported rarely prescribing behavioral therapy for people with dementia, but 11 had never done so before. Behavioral therapy was mainly associated with mental disorders (*n* = 5), for example, phobia (*n* = 3), behavior (*n* = 2), and the evidence base (*n* = 1). One practitioner reported that behavioral therapy had not been relevant during medical studies, seven said that it did not matter much, and four reported that it did matter somewhat. One practitioner reported daily experience with behavioral therapy, and two reported almost no experience. Mainly, there were positive views on behavioral therapy (*n* = 8), for example, regarding the pragmatism of the approach (*n* = 2). However, there was also criticism, for example, related to the supply, “*when it comes to psychotherapy, people get frustrated very quickly because they cannot reach anyone and do not get a place*,” the nature of behavioral therapy, “*manuals often do not do justice to the more complex reality*” and the training, “*what I blame behavior therapy for, in its educational context, is that, in my opinion, it contains too little self-experience*.” Patients’ reactions to behavioral therapy were said to be mainly positive (*n* = 7) but also sometimes skeptical, for example, due to prejudices (*n* = 2), the supply situation (*n* = 2), or because they expect the process to be more passive and less straining (*n* = 1). Even though behavioral therapy cannot be prescribed, as four participants stated, six practitioners reported having recommended behavioral therapy in the past, five of whom on a regular basis. When asked about dementia, 10 practitioners answered that they had never recommended behavioral therapy for people with dementia, and their opinions were mostly skeptical. One participant said: “*Nope, that does not lead anywhere. That’s rubbish.*”

#### Treatment

When asked for suitable treatment for people with dementia, the general practitioners listed activities (*n* = 4), exercises (*n* = 4), and physiotherapy (*n* = 2). Three mentioned pharmacological treatment (while four others did not think pharmacological therapy was effective for treating dementia) and cognitive training (*n* = 3). One each listed activation in groups, modification of daily life, routines, communication, animal-assisted approaches, healthy nutrition and nutritional supplements, and supply of sufficient fluids. At the end of the interview, three participants also mentioned occupational therapy and one behavioral therapy. One practitioner argued that it is about the care itself but not about a certain approach: “O*ccupational therapy is what is most likely to be prescribed. But in the end, anyone, any FSJ* [a year of voluntary social service] *student can go and take them for a walk. The patients think that is just as good.*” One participant concluded: “*Well, the real art is to find a fit.*” Additionally, the importance of the home environment for the person with dementia was expressed: “*I think, it would be so much nicer and more helpful for the people, if they could just stay in the privacy of their home*.” Therefore, “*above all, it would have to be an outreach offer*,” but at the same time, “*when they have to be visited, it gets a little more difficult*.” Additionally, for the diagnosis, home visits might be helpful: “*Especially during home visits, you notice this faster*.”

As suitable treatments for family caregivers, practitioners enumerate support groups (*n* = 8), information or advice offers (*n* = 5), relief offers (*n* = 5), psychological support (*n* = 4, while two other practitioners said this was not necessary), and primary care (*n* = 1).

#### Family caregivers of people with dementia

Family caregivers were described as overstrained and desperate (*n* = 5), overambitious (*n* = 1), but also as grateful (*n* = 3). Additionally, guilt was perceived as a central feeling connected to caregiving. Another problem mentioned multiple times is the growing distance between relatives due to work-related mobility. The collaboration of practitioners with caregivers was described variably, mostly as support (*n* = 5) but also as an additional task (*n* = 3). One interviewee said that family caregivers were “*often a support, sometimes the only challenge.”* According to the participants, most caregivers desired relief (*n* = 5), support (*n* = 3), and information (*n* = 2), and some also asked for medication. If the caregiver is the patient’s partner, they usually want to stay together at home (*n* = 1). Central concerns beyond being overstrained were helplessness (*n* = 2) and fear (*n* = 1).

#### Interdisciplinary exchange

The practitioners tended to welcome interdisciplinary exchange: “*The nice thing about it is that you talk about it and learn a lot about the patient through this interprofessional dialog and, of course, learn about the other profession as well.*” One practitioner gave the advice to “*build in a feedback loop*.” It was criticized that there are few opportunities for structured and paid exchange: “*But actually, in my opinion, we have to come to the point in healthcare that there are paid time slots for interprofessional dialog*”/ “*Every phone call I make is in my spare time.*” Another advice was to arrange convenient times for such meetings.

The expectations for documentation varied widely. This is probably also due to the different reporting behaviors: “*There are people who can keep things short and people who do not write anything in a long, long [report].*” Again, most practitioners preferred written documentation (*n* = 10). They argued that the written form was best for the filing (*n* = 2) as well as for their time-management (*n* = 2). Only the written form via e-mail was criticized due to concerns regarding data protection. Four welcomed the personal exchange: “*For me, the brief contact with the therapist is more important than a written report*” and “*rather a short conversation than a long report*.” Another four had concerns, however, including time constraints and doubts about the need. Three welcomed an exchange by telephone, while another three did not like the idea, one of which noted that you need to agree on a time for the call. One practitioner differentiated between occupational (“*a letter is sufficient*”) and behavioral therapy (“*a letter with an additional visit and call*”). The expectations for the content were consensual, however: “*I need this information: Where are the resources, where are the deficits, and how can you strengthen the resources and better compensate or eliminate the deficits?*” Most practitioners would be content receiving a summarized report at the end of the prescribed therapy (*n* = 8). Nine practitioners wanted to be informed about the treatment progress, eight appreciated recommendations regarding further proceedings, four were interested in observations made by the therapists, and three also wanted to know more about the content of the sessions. One practitioner added that she would also like to use some space on the letter of referral to take notes herself. And an additional aspect of an unmet documentation need was identified: “*Patients […] give you constant feedback, and that is of course partly very interesting. Sometimes I would wish that I had a more structured process for processing this information.*”

#### Obstacles

Six practitioners perceived budgeting as a limitation to prescribing occupational therapy. Three described budgeting as a threat, and one practitioner called it the “*sword of Damocles*.” Five participants indicated unfamiliarity with the regulations regarding extrabudgetary prescriptions and special needs supplies: “*For example, I do not really know—that is, of course, again due to the lack of experience with occupational therapy—whether that is also part of the physiotherapy budget.*”/ “*You are grateful for every prescription that you do not have to write yourself.*” Only two practitioners referred to the extrabudgetary prescription of occupational therapy: “*Whereby dementia thankfully for a bunch of indications, including above all occupational therapy, can be prescribed in such a way that it is harmless as a prescription of the special remedy needs. Which, by the way, I do not think many people know, which of course is a shame.*” As another obstacle to prescribing occupational therapy, a lack of knowledge was named: “*Yes, of course also not knowing exactly what the options are and not having it in the back of your mind.*” Regarding behavioral therapy, one practitioner argued: “*I would have doubts that dementia will be recognized as a prognostically influenceable disorder.*”

#### Education

The practitioners wished for further education on non-pharmacological interventions for dementia. The limited offer was criticized: “*Dealing with and training on the subject of dementia could be better*” and “*physiotherapy and occupational therapy—there is no offer at all. At least not in primary care.*” One participant concluded, “*You are never informed enough. Or there is so much good [supply] that you do not know about. But this is not a dementia-specific problem.*”

#### Further criticism

Some additional obstacles in the health care supply for people with dementia were expressed, including being dependent on the family for information on the patient, a lack of prevention, a lack of possibilities for health care at home, a lack of resources (e.g., specialized care stations or day care), and societal stigmatization. Another interesting aspect was criticism of care services regarding irregular visiting times, forcing patients to spend hours waiting instead of being active. General frustration regarding the current health care supply became apparent in the statement: “*What improves care is not intended in the care.*” Regarding the family caregivers, practitioners criticized the small amount of relief offers and financial support.

## Discussion

The combination of quantitative and qualitative approaches provided insight into the practice of health care regarding the treatment of dementia and underlying attitudes. Two online surveys with medical students and doctors showed a lack of familiarity with non-pharmacological interventions such as occupational and behavioral therapy, especially as treatments for individuals with dementia and their caregivers. A third online survey revealed that general practitioners predominantly prescribed pharmacological treatment for people with dementia. At the same time, the practitioners expected non-pharmacological interventions to be more effective than medical treatment for MCI or mild dementia. Interviews with general practitioners revealed diverse attitudes toward occupational and behavioral therapy, but uncertainty regarding budgeting and a pessimistic view of dementia became apparent. Within the questions, “People with dementia” was not further specified. This might be a limitation and should be considered when interpreting the results. A precise description of a specific patient group might have led to different answers. This could be modified in further investigations or participants could be asked what they are thinking about in terms of disease stages and symptoms.

### Study survey

The student sample comprised 115 students drawn from a population of about 113,383 medical students (47) from different years of study, which means that their knowledge differed widely. Also, there was no control for gender, as an effect of gender on this topic was not expected and the survey should be kept short. The answers suggest that the coverage of occupational and behavioral therapy in medical studies, especially as a treatment for individuals with dementia, does not match their status as effective non-pharmacological treatments in the treatment guidelines. The expected content was similar to that of the general public and that of occupational and behavioral therapists, as reported in Frankenstein and Jahn (6). To improve healthcare, medical studies need to be informed about secondary symptoms of dementia, effective treatment, and non-pharmacological interventions to slow progression, to foster autonomy, to treat secondary symptoms, and to improve quality of life of individuals living with dementia and their caregivers.

### Knowledge survey

The survey results obtained from practitioners (*n* = 19 drawn from a population of about 569,000 practitioners (48)) confirmed the former results regarding the low coverage of non-pharmacological interventions in medical studies. Furthermore, it became evident that there was a mismatch between the frequency of prescriptions or recommendations for behavioral and occupational therapy and treatment guidelines. Clearly, better informing practitioners about non-pharmacological therapy options, treatment guidelines, and regulations for prescription is necessary to provide effective treatment.

### Prescription survey

Practitioners were more familiar with medical therapy for people with dementia: The least-known drug category “antidepressants” was on the same level as the best-known non-pharmacological intervention “occupational therapy.” However, practitioners expected non-pharmacological interventions to be generally more effective than medication for people with MCI or mild dementia in terms of cognition, daily functioning, quality of life, and behavior. Nevertheless, only half of the participating practitioners were aware of the option of an extrabudgetary prescription of occupational therapy. Again, there is an obvious need for additional information, especially considering that antidepressants and antipsychotics might negatively impact cognitive functioning and can accelerate degradation (49, 50).

### Interviews with general practitioners

The interviews were intended to be qualitative explorations of the beliefs, experiences, and thoughts of general practitioners about dementia, behavioral therapy, and occupational therapy. One major barrier to making use of non-pharmacological interventions was uncertainty regarding budgeting. It seems necessary to clearly communicate that prescribing occupational therapy does not strain the assigned budget. The knowledge about this opportunity might have increased with the legal validity of the 2021 updated therapy guideline (28), which forced general practitioners to engage in new requirements for prescriptions. Nevertheless, more information on extrabudgetary prescriptions from sources perceived as reliable by practitioners would probably positively influence the health care supply. Additionally, a one-sided view on non-pharmacological interventions became apparent. For example, some practitioners did not picture occupational therapy as a structured approach toward maximal independence. Here, as well, general practitioners need to be better informed, and additional high-quality evidence needs to be generated. A blank prescription could be a valuable opportunity to assign specific treatment decisions to the therapists.

### Limitations

A general limitation of online surveys is an uncertainty regarding the accuracy of the information, especially the demographic information, provided by the participants. None of the samples are representative, as the invitations were not evenly spread across the entire populations, for example, demographic and individual characteristics, such as sex and age, were not balanced. Moreover, the second survey and the interviews comprised only a small sample size. Additionally, the participating practitioners might be biased regarding their attitudes, for example, by being generally more interested in research, dementia, or non-pharmacological interventions, and therefore might have been more open or better informed than practitioners who could not be recruited. Still, valuable insight into the education and practical dimensions of non-pharmacological treatment, such as occupational and behavioral therapy for people with dementia, was gained.

### Supply situation

In Germany, there are about 48,000 psychotherapists, 60% of which work in the health supply system (17). Dementia is not a diagnosis that justifies psychotherapy, but the treatment of secondary symptoms, such as depression or anxiety can be covered. The use of psychotherapy among people aged 70–79 years was still at 61% of those with self-reported mental health problems, while the highest use was found in the age group 18–29 (72%) and the lowest (55%) in the age group 30–39. The mean waiting time between request and guideline psychotherapy is 19.6 weeks (10.5 weeks for acute therapy) (17).

There were 10,399 occupational therapy practices in 2022. Of all occupational therapy prescriptions registered by the health insurance AOK in 2022, 4.4% have been delivered to people with dementia. In adulthood the amount of occupational therapy provided increases with age (e.g., 926 treatments for patients 85–89 years old, 983 treatments for patients above 90 years of age). Occupational therapy is most frequently prescribed by general practitioners (51). It can also be prescribed extrabudgetarily for people with dementia that are over 70 years old and for patients with early-onset dementia (28) and since 2021 psychological psychotherapists can also prescribe occupational therapy.

The” S3 Leitlinie Demenzen” (S3 guideline dementia) comprises recommendations for optimal care. It suggests using cognitive stimulation, reminiscence therapy, or occupational therapy to address depression in dementia (3). Since 2020, the National Dementia Strategy (“Nationale Demenzstrategie”) is in force and aims to improve the situation of people with dementia and their caregivers (52). If occupational or behavioral therapy for people with dementia were suddenly to be prescribed regularly, it would certainly be a challenge to meet the demand, but in the long term, it should be possible to adapt.

A similar situation is described by Ayeno et al. regarding the use of non-pharmacological therapies for the management of BPSD in Australian care residents with dementia (31). Of the 96 participants (physicians, nurses and paid caregivers) 66% agreed, that “non-pharmacological interventions are more useful than medication for management of BPSD.” Most participants were familiar with non-pharmacological interventions, most frequently used were redirection, behavior management and validation. But 84% agreed, that there were “insufficient human resources” for non-pharmacological interventions (31).

## Conclusion

Two major obstacles in the supply of non-pharmacological interventions for people with dementia were observed: A lack of knowledge about approaches such as behavioral and occupational therapy reduces the likelihood of making use of these offers, and a lack of secured knowledge on prescription and budgeting guidelines causes further uncertainty. Additionally, interdisciplinary exchange is hampered by time constraints and the workload of practitioners, as well as by missing compensation. Uncovering these barriers is highly important for overcoming them in the future.

### Perspective

To improve the standard of care for people with dementia and their family caregivers, barriers to the use of effective non-pharmacological interventions such as behavioral and occupational therapy need to be overcome. More comprehensive evaluation studies on behavioral and occupational therapy with well-trained study therapists are needed. Behavioral therapy should also be considered for people with dementia and covered by health insurance where necessary. A highly pressing further education goal should be to improve the understanding and comprehensibility of budgetary regulations, including the possibility of an extrabudgetary prescription of occupational therapy for people with dementia. It is highly important to better inform general practitioners about non-pharmacological therapy options and to bridge the gap between therapists and general practitioners. According to Schoenmakers et al., interventions to improve general practitioners’ skills are appropriate for expanding awareness and knowledge, but the effects are limited (35). Preferably, basic knowledge on non-pharmacological interventions for people with dementia and their family caregivers should be gained during medical studies. Collecting and providing information was started and it can be found on this website: https://mytuc.org/nwbx.

## Data Availability

The datasets presented in this study can be found in online repositories. The names of the repository/repositories and accession number(s) can be found at: https://osf.io/g2a4q/.
