# Supplementary figures and images for: General practitioners’ and medical students’ current knowledge and attitudes toward non-pharmacological interventions for dementia
Source: Front Med (Lausanne). 2025 Jul 23;12:1573251. doi: 10.3389/fmed.2025.1573251 (PMC12325421; doi:10.3389/fmed.2025.1573251)

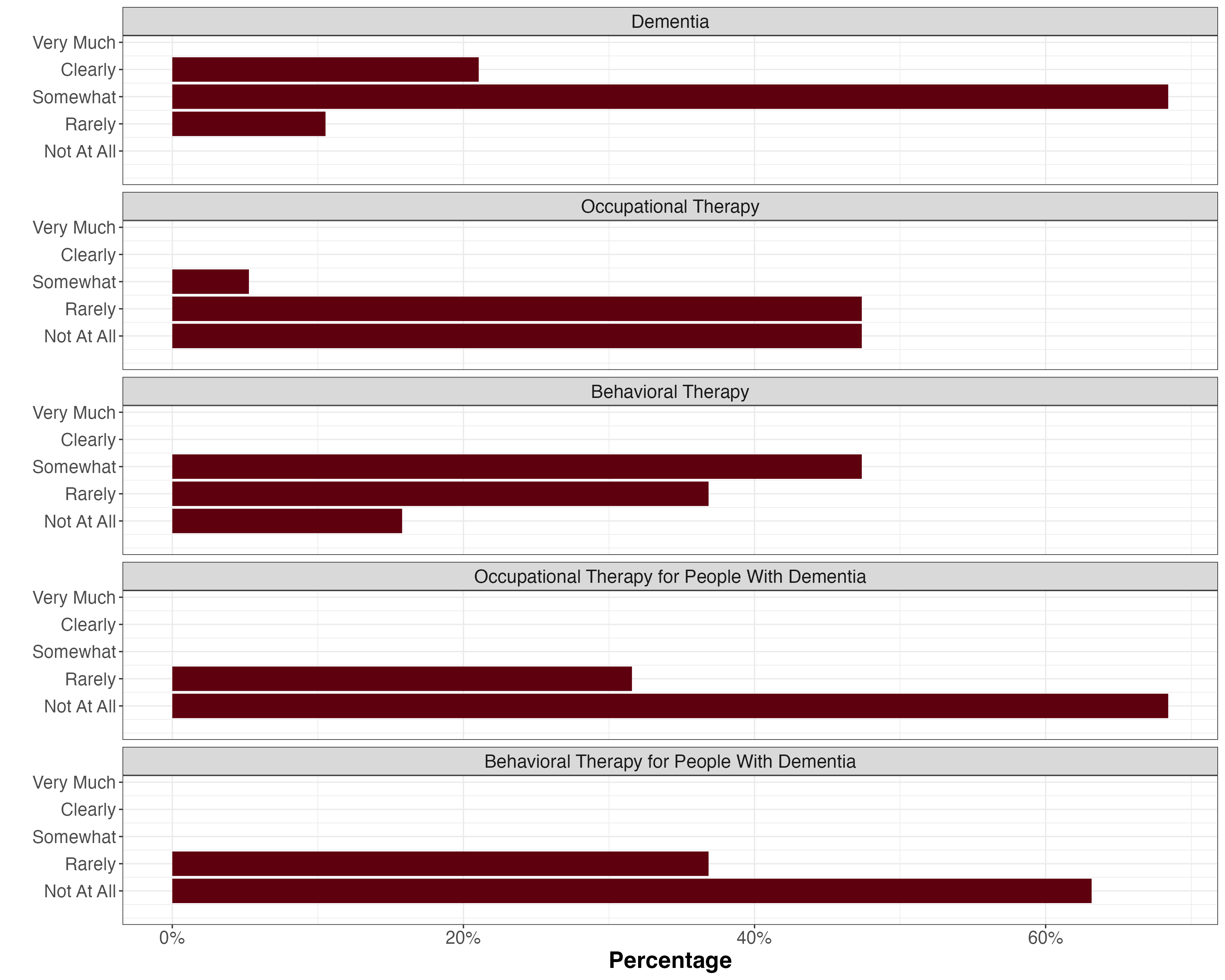

Supplement: Supplementary file 1 [file Image_1.TIFF]
