# Supplementary material for: General practitioners’ and medical students’ current knowledge and attitudes toward non-pharmacological interventions for dementia
Source: Front Med (Lausanne). 2025 Jul 23;12:1573251. doi: 10.3389/fmed.2025.1573251 (PMC12325421; doi:10.3389/fmed.2025.1573251)
Supplement: Supplementary file 2 [file Data_Sheet_1.pdf]

soSci
oFb - der onlineFragebogen

ETVTStudierende → base 28.12.2023, 16:22

Korrekturfahne

Bitte beachten Sie, dass Filter und Platzhalter in der Druckansicht prinzipbedingt nicht funktionieren. Fragen, die mittels PHP-Code eingebunden sind, werden nur eingeschränkt wiedergegeben.

Bitte beachten Sie folgende Unterschiede zum tatsächlichen Fragebogen:

- Filter können prinzipbedingt nicht funktionieren,
- Fragen im PHP-Code werden nur angezeigt, wenn die Kennung statisch vorliegt,
- die Anzeige der Fragen kann abweichen, weil die Frage-Kennungen eingeblendet werden, und
- Platzhalter und andere dynamische Elemente können prinzipbedingt nicht dargestellt werden.

Tipp: Stellen Sie in den Druck-Einstellungen Ihres Browser ein, dass dieser auch Hintergrundbilder druckt, damit auch Schieberegler und benutzerdefinierte Eingabefelder korrekt gedruckt bzw. in ein PDF übernommen werden.

- ☑ Kennungen & Notizen
- ☑ Filter
- ☐ Variablen
- ☑ PHP-Code
- ☐ HTML-Elemente
- ☐ JavaScript
- **Tabelle (Download)**
- **Seite drucken / PDF**

Seite 01

Einleitung

Ich bin Diplom-Psychologin und promoviere am Lehrstuhl für Angewandte Gerontopsychologie und Kognition an der Technischen Universität Chemnitz. Ziel meiner Arbeit ist die Verbesserung der Versorgung von Menschen mit Demenz und ihrer Angehörigen.

Für einen Einblick in die Praxis der Versorgung und Ausbildung bitte ich Sie um die Beantwortung einiger weniger Fragen und freue mich über Hinweise zu diesem Thema aus Ihrer Sicht. Weil ich weiß, dass Sie als Medizinstudierende ein hohes Arbeitspensum zu bewältigen haben und die aktuelle Situation zusätzliche Anforderungen an Sie stellt, habe ich den Umfang auf das Allernötigste (ca. 5 Minuten) beschränkt.

Ihre Angaben bleiben anonym und werden nur für wissenschaftliche Zwecke verwendet. Für Fragen und Anregungen stehe ich Ihnen selbstverständlich gerne zur Verfügung: lou.frankenstein@s2018.tu-chemnitz.de

Herzlichen Dank für Ihre Unterstützung!

Seite 02

DM

1. Bitte nennen Sie Ihren Studiengang.

DM05 Studiengang

2. In welchem Semester befinden Sie sich derzeit?

DM03 Semester

- ○ 1
- ○ 2
- ○ 3
- ○ 4
- ○ 5
- ○ 6
- ○ 7
- ○ 8
- ○ 9
- ○ 10
- ○ 11
- ○ 12
- ○ 13
- ○ 14
- ○ 15
- ○ 16
- ○ 17
- ○ 18
- ○ 19
- ○ 20
- ○ mehr als 20

3. In welchem Bundesland studieren Sie?

DM02 Bundesland

- ○ Baden-Württemberg
- ○ Bayern
- ○ Berlin
- ○ Brandenburg
- ○ Bremen
- ○ Hamburg
- ○ Hessen
- ○ Mecklenburg-Vorpommern
- ○ Niedersachsen
- ○ Nordrhein-Westfahlen
- ○ Rheinland Pfalz
- ○ Saarland
- ○ Sachsen
- ○ Sachsen Anhalt
- ○ Schleswig Holstein
- ○ Thüringen

4. An welcher Universität studieren Sie?

DM04 Universität

Seite 03

ET

5. Was kommt Ihnen als Erstes in den Sinn, wenn Sie an Ergotherapie denken?

ET04 Assoziationen ET

1.
2.
3.

6. Ergotherapie bei Demenz ist...

ET05 Schieberegler ET

unstrukturiert	strukturiert
nicht wissenschaftlich	wissenschaftlich
schwer zugänglich	niedrigschwellig
individuell	allgemein
erniedrigend	wertschätzend
ungesellig	sozial
theoretisch	praktisch
nutzlos	nützlich
unwichtig	wichtig
langweilig	herausfordernd
beängstigend	ermutigend
unangenehm	angenehm
unterschätzt	überschätzt

7. Welche Inhalte sind Ihres Wissens nach Teil einer ergotherapeutischen Behandlung (hier sind mehrere Antworten möglich)?

ET06 Inhalt ET

- ☐ Diagnostik
- ☐ alltägliche Abläufe / Handlungen
- ☐ Verhalten
- ☐ Selbstständigkeit
- ☐ Kommunikation
- ☐ Wohnraumanpassung
- ☐ Hilfsmittel
- ☐ Psychoedukation
- ☐ Lebensrückblick
- ☐ körperliche Aktivität
- ☐ Denkaufgaben
- ☐ basteln / werken

Seite 04

VT

8. Was kommt Ihnen als Erstes in den Sinn, wenn Sie an Verhaltenstherapie denken?

VT04 Assoziationen VT

1.
2.
3.

9. Verhaltenstherapie bei Demenz ist...

VT05 Schieberegler VT

unstrukturiert	strukturiert
nicht wissenschaftlich	wissenschaftlich
schwer zugänglich	niedrigschwellig
individuell	allgemein
erniedrigend	wertschätzend
ungesellig	sozial
theoretisch	praktisch
nutzlos	nützlich
unwichtig	wichtig
langweilig	herausfordernd
beängstigend	ermutigend
unangenehm	angenehm
unterschätzt	überschätzt

10. Welche Inhalte sind Teil einer verhaltenstherapeutischen Behandlung (hier sind mehrere Antworten möglich)?

VT06 Inhalt VT

- ☐ Diagnostik
- ☐ alltägliche Abläufe / Handlungen
- ☐ Verhalten
- ☐ Selbstständigkeit
- ☐ Kommunikation
- ☐ Wohnraumanpassung
- ☐ Hilfsmittel
- ☐ Psychoedukation
- ☐ Lebensrückblick
- ☐ körperliche Aktivität
- ☐ Denkaufgaben
- ☐ basteln / werken

Seite 05

Inhalte

11. Wie sehr waren die folgenden Inhalte Teil Ihres Studiums?

IN01 Studieninhalte

	überhaupt nicht	kaum	etwas	deutlich	sehr
Ergotherapie	○	○	○	○	○
Verhaltenstherapie	○	○	○	○	○
Demenz	○	○	○	○	○
Ergotherapie bei Demenz	○	○	○	○	○
Verhaltenstherapie bei Demenz	○	○	○	○	○

12. Sollten Sie einen allgemeinen Kommentar oder einen Hinweis haben, können Sie diesen hier notieren:

ST01 Kommentar

Letzte Seite

Herzlichen Dank für Ihre Teilnahme!

Ihre Antworten wurden gespeichert, Sie können das Browser-Fenster nun schließen.

Möchten Sie in Zukunft an interessanten und spannenden Online-Befragungen teilnehmen?

Wir würden uns sehr freuen, wenn Sie Ihre E-Mail-Adresse für das SoSci Panel anmelden und damit wissenschaftliche Forschungsprojekte unterstützen.

E-Mail: Am Panel teilnehmen

Die Teilnahme am SoSci Panel ist freiwillig, unverbindlich und kann jederzeit widerrufen werden.
Das SoSci Panel speichert Ihre E-Mail-Adresse nicht ohne Ihr Einverständnis, sendet Ihnen keine Werbung und gibt Ihre E-Mail-Adresse nicht an Dritte weiter.

Sie können das Browserfenster selbstverständlich auch schließen, ohne am SoSci Panel teilzunehmen.
