# Supplementary material for: General practitioners’ and medical students’ current knowledge and attitudes toward non-pharmacological interventions for dementia
Source: Front Med (Lausanne). 2025 Jul 23;12:1573251. doi: 10.3389/fmed.2025.1573251 (PMC12325421; doi:10.3389/fmed.2025.1573251)
Supplement: Supplementary file 3 [file Data_Sheet_2.pdf]

soSci
oFb - der onlineFragebogen

ETVT → base

28.12.2023, 16:21

Korrekturfahne

Bitte beachten Sie, dass Filter und Platzhalter in der Druckansicht prinzipbedingt nicht funktionieren. Fragen, die mittels PHP-Code eingebunden sind, werden nur eingeschränkt wiedergegeben.

Bitte beachten Sie folgende Unterschiede zum tatsächlichen Fragebogen:

- Filter können prinzipbedingt nicht funktionieren,
- Fragen im PHP-Code werden nur angezeigt, wenn die Kennung statisch vorliegt,
- die Anzeige der Fragen kann abweichen, weil die Frage-Kennungen eingeblendet werden, und
- Platzhalter und andere dynamische Elemente können prinzipbedingt nicht dargestellt werden.

Tipp: Stellen Sie in den Druck-Einstellungen Ihres Browser ein, dass dieser auch Hintergrundbilder druckt, damit auch Schieberegler und benutzerdefinierte Eingabefelder korrekt gedruckt bzw. in ein PDF übernommen werden.

- ☑ Kennungen & Notizen
- ☑ Filter
- ☐ Variablen
- ☑ PHP-Code
- ☐ HTML-Elemente
- ☐ JavaScript
- **Tabelle (Download)**
- **Seite drucken / PDF**

Seite 01

Ich bin Diplom-Psychologin und promoviere am Lehrstuhl für Angewandte Gerontopsychologie und Kognition an der Technischen Universität Chemnitz. Ziel meiner Arbeit ist die Verbesserung der Versorgung von Menschen mit Demenz und ihrer Angehörigen.

Um einen Einblick in die Versorgung zu erhalten, bitte ich Sie um die Beantwortung einiger weniger Fragen und freue mich über Hinweise zu diesem Thema aus Ihrer Sicht. Weil ich weiß, dass Sie als Mediziner_in ein hohes Arbeitspensum zu bewältigen haben und die aktuelle Situation zusätzliche Anforderungen an Sie stellt, habe ich den Umfang auf das Allernötigste (ca. 3 Minuten) beschränkt.

Ihre Angaben bleiben anonym und werden nur für wissenschaftliche Zwecke verwendet. Für Fragen und Anregungen stehe ich Ihnen selbstverständlich gerne zur Verfügung: lou.frankenstein@s2018.tu-chemnitz.de

Herzlichen Dank für Ihre Unterstützung!

Seite 02

Demo

1. Bitte nennen Sie Ihre genaue Berufsbezeichnung.

DM19 Beruf

2. Wie alt sind Sie?

DM17 Alter

- ○ 20 – 29 Jahre alt
- ○ 30 – 39 Jahre alt
- ○ 40 – 49 Jahre alt
- ○ 50 – 59 Jahre alt
- ○ 60 – 69 Jahre alt
- ○ 70 Jahre und älter

3. Bitte nennen Sie Ihr Geschlecht.

DM18 Geschlecht

- ○ weiblich
- ○ männlich
- ○ divers

4. In welchem Bundesland haben Sie (vorrangig) studiert?

DM20 Bundesland S

- ○ Baden-Württemberg
- ○ Bayern
- ○ Berlin
- ○ Brandenburg
- ○ Bremen
- ○ Hamburg
- ○ Hessen
- ○ Mecklenburg-Vorpommern
- ○ Niedersachsen
- ○ Nordrhein-Westfalen
- ○ Rheinland-Pfalz
- ○ Saarland
- ○ Sachsen
- ○ Sachsen-Anhalt
- ○ Schleswig-Holstein
- ○ Thüringen

5. In welchem Bundesland sind Sie (vorrangig) tätig?

DM21 Bundesland A

- ○ Baden-Württemberg
- ○ Bayern
- ○ Berlin
- ○ Brandenburg
- ○ Bremen
- ○ Hamburg
- ○ Hessen
- ○ Mecklenburg-Vorpommern
- ○ Niedersachsen
- ○ Nordrhein-Westfalen
- ○ Rheinland-Pfalz
- ○ Saarland
- ○ Sachsen
- ○ Sachsen-Anhalt
- ○ Schleswig-Holstein
- ○ Thüringen

Seite 03

Praxis

6. Wie sehr waren die folgenden Inhalte Teil Ihres Studiums?

IN01 Studieninhalte

	überhaupt nicht	kaum	etwas	deutlich	sehr
Ergotherapie	○	○	○	○	○
Verhaltenstherapie	○	○	○	○	○
Demenz	○	○	○	○	○
Ergotherapie bei Demenz	○	○	○	○	○
Verhaltenstherapie bei Demenz	○	○	○	○	○

7. Wie häufig verschreiben oder empfehlen Sie

P101 Verschreibung

	nie	selten	manchmal	häufig	sehr häufig
Ergotherapie, allgemein	○	○	○	○	○
Ergotherapie bei Vorliegen einer Demenz	○	○	○	○	○
Verhaltenstherapie allgemein	○	○	○	○	○
Verhaltenstherapie bei Vorliegen einer Demenz	○	○	○	○	○

8. Was könnte Sie dazu veranlassen, mehr Ergo- und Verhaltenstherapie bei Demenz zu verschreiben?

P102 ET/VT

Seite 04

Doku

9. Welche Informationen über die Patient_innen und den Behandlungsfortgang benötigen Sie?

D102 Informationen

- ☐ eine Zusammenfassung jeder Sitzung
- ☐ Information über Zwischenerfolge oder Rückschritte
- ☐ physische Auffälligkeiten
- ☐ psychische Auffälligkeiten
- ☐ Empfehlungen hinsichtlich der weiteren Behandlung
- ☐ Abschlussbericht
- ☐ weitere

10. Wenn Sie weitere Informationen benötigen, welche sind das?

D104 weitere Informationen

11. Auf welchem Wege möchten Sie diese Informationen erhalten?

D101 Weg

- ☐ persönlich, bei einem Treffen
- ☐ telefonisch
- ☐ schriftlich
- ☐ anderer Weg

12. Wenn Sie die Informationen auf anderem Weg erhalten möchten, welcher ist das?

D103 anderer Weg

13. Haben Sie weitere Anmerkungen oder Hinweise zu nicht-medikamentösen Interventionen bei Demenz, interdisziplinärem Austausch oder der Zusammenarbeit mit Mediziner_innen?

S101 Kommentar

Letzte Seite

Herzlichen Dank für Ihre Teilnahme!

Ihre Antworten wurden gespeichert, Sie können das Browser-Fenster nun schließen.

Möchten Sie in Zukunft an interessanten und spannenden Online-Befragungen teilnehmen?

Wir würden uns sehr freuen, wenn Sie Ihre E-Mail-Adresse für das SoSci Panel anmelden und damit wissenschaftliche Forschungsprojekte unterstützen.

E-Mail: [ ] Am Panel teilnehmen

Die Teilnahme am SoSci Panel ist freiwillig, unverbindlich und kann jederzeit widerrufen werden.
Das SoSci Panel speichert Ihre E-Mail-Adresse nicht ohne Ihr Einverständnis, sendet Ihnen keine Werbung und gibt Ihre E-Mail-Adresse nicht an Dritte weiter.

Sie können das Browserfenster selbstverständlich auch schließen, ohne am SoSci Panel teilzunehmen.
