# Supplementary material for: General practitioners’ and medical students’ current knowledge and attitudes toward non-pharmacological interventions for dementia
Source: Front Med (Lausanne). 2025 Jul 23;12:1573251. doi: 10.3389/fmed.2025.1573251 (PMC12325421; doi:10.3389/fmed.2025.1573251)
Supplement: Supplementary file 4 [file Data_Sheet_3.pdf]

Forschungsarbeit zur medikamentösen und nicht-medikamentösen Therapie bei Demenz

Herzlich Willkommen und vielen Dank vorab für Ihr Interesse an diesem Forschungsprojekt.
Ich interessiere mich für die Unterschiede zwischen medikamentösen und nicht-medikamentösen Verfahren zur Behandlung von Menschen mit Demenz.
Hierfür möchte ich Sie darum bitten an dieser ca. 10-20 minütigen Umfrage teilzunehmen. Es gibt keine falschen Antworten. Daher bitte ich Sie, die Fragen frei nach Ihrer eigenen Meinung zu beantworten.
Bei Fragen können Sie mich gerne per E-Mail, unter folgender Adresse, kontaktieren:
Lea Pickard: lea.pickard@gmx.de

Die Richtlinien guter ethischer Forschung sehen vor, dass sich die Teilnehmer/innen an empirischen Studien explizit und nachvollziehbar mit der Teilnahme einverstanden erklären.

Freiwilligkeit. Ihre Teilnahme an dieser Untersuchung ist freiwillig. Es steht Ihnen zu jedem Zeitpunkt dieser Umfrage frei, Ihre Teilnahme abzubrechen, ohne dass Ihnen daraus Nachteile entstehen.

Anonymität. Ihre Daten sind selbstverständlich vertraulich, werden nur in anonymisierter Form ausgewertet und nicht an Personen, die nicht am Forschungsprojekt beteiligt sind, weitergegeben. Demographische Angaben wie Alter oder Geschlecht lassen keinen eindeutigen Schluss auf Ihre Person zu.

Fragen. Falls Sie noch Fragen zu dieser Umfrage haben sollten, können Sie sich gerne per E-Mail an Lea Pickard, deren E-Mail-Adresse Sie weiter oben finden, wenden.

Hiermit bestätige ich, dass ich mindestens 18 Jahre alt bin sowie die Einverständniserklärung gelesen und verstanden habe.

- ○ Nein (nicht an der Studie teilnehmen)
- ○ Ja

1. Wie alt sind Sie?

2. Welchem Geschlecht fühlen Sie sich zugehörig?

[Bitte auswählen]

3. In welchem medizinischen Fachbereich sind Sie tätig?

- ○ Psychiatrie
- ○ Allgemeinmedizin
- ○ Neurologie
- ○ Sonstiges

4. In welchem Jahr haben Sie das Medizinstudium beendet?

5. In welchem Bundesland sind Sie tätig?

[Bitte auswählen]

6. Welche der folgenden Medikamente zur Behandlung von Demenz kennen Sie?

- ☐ Rivastigmin
- ☐ Galantamin
- ☐ Donepezil
- ☐ Memantin
- ☐ Antidepressiva
- ☐ Antipsychotika
- ☐ Sonstige ____________

7. Wie häufig verschreiben Sie, bei Patient:innen mit einer Demenzerkrankung, Medikamente zur Behandlung von Demenz?

○	○	○	○	○	○
**nie**	**< 25%** der Patient:innen	**< 50%** der Patient:innen	**> 50%** der Patient:innen	**> 75%** der Patient:innen	**immer**

8. Wie gut ist Ihrer Meinung nach die Wirkung von Memantin auf die kognitiven Fähigkeiten Ihrer Patient:innen?

	stark negative Wirkung						stark positive Wirkung	kann ich nicht beurteilen
bei leichten kognitiven Einschränkungen	○	○	○	○	○	○	○	○
bei leichter Demenz	○	○	○	○	○	○	○	○
bei mittelschwerer Demenz	○	○	○	○	○	○	○	○
bei schwerer Demenz	○	○	○	○	○	○	○	○

9. Wie gut ist Ihrer Meinung nach die Wirkung von Memantin auf die Lebensqualität und die Aktivitäten des täglichen Lebens Ihrer Patient:innen?

	stark negative Wirkung						stark positive Wirkung	kann ich nicht beurteilen
bei leichten kognitiven Einschränkungen	○	○	○	○	○	○	○	○
bei leichter Demenz	○	○	○	○	○	○	○	○
bei mittelschwerer Demenz	○	○	○	○	○	○	○	○
bei schwerer Demenz	○	○	○	○	○	○	○	○

10. Wie gut ist Ihrer Meinung nach die Wirkung von Memantin auf die Verhaltensparameter Ihrer Patient:innen?

mit Verhaltensparametern sind in diesem Kontext Begleiterscheinungen, wie Depressionen, Wut, Ärger, Psychosen, etc. zu verstehen

	stark negative Wirkung						stark positive Wirkung	kann ich nicht beurteilen
bei leichten kognitiven Einschränkungen	○	○	○	○	○	○	○	○
bei leichter Demenz	○	○	○	○	○	○	○	○
bei mittelschwerer Demenz	○	○	○	○	○	○	○	○
bei schwerer Demenz	○	○	○	○	○	○	○	○

11. Wie gut ist Ihrer Meinung nach die Wirkung von Rivastigmin auf die kognitiven Fähigkeiten Ihrer Patient:innen?

	stark negative Wirkung						stark positive Wirkung	kann ich nicht beurteilen
bei leichten kognitiven Einschränkungen	○	○	○	○	○	○	○	○
bei leichter Demenz	○	○	○	○	○	○	○	○
bei mittelschwerer Demenz	○	○	○	○	○	○	○	○
bei schwerer Demenz	○	○	○	○	○	○	○	○

12. Wie gut ist Ihrer Meinung nach die Wirkung von Rivastigmin auf die Lebensqualität und die Aktivitäten des täglichen Lebens Ihrer Patient:innen?

	stark negative Wirkung						stark positive Wirkung	kann ich nicht beurteilen
bei leichten kognitiven Einschränkungen	○	○	○	○	○	○	○	○
bei leichter Demenz	○	○	○	○	○	○	○	○
bei mittelschwerer Demenz	○	○	○	○	○	○	○	○
bei schwerer Demenz	○	○	○	○	○	○	○	○

13. Wie gut ist Ihrer Meinung nach die Wirkung von Rivastigmin auf die Verhaltensparameter Ihrer Patient:innen?

mit Verhaltensparametern sind in diesem Kontext Begleiterscheinungen, wie Depressionen, Wut, Ärger, Psychosen, etc. zu verstehen

	stark negative Wirkung						stark positive Wirkung	kann ich nicht beurteilen
bei leichten kognitiven Einschränkungen	○	○	○	○	○	○	○	○
bei leichter Demenz	○	○	○	○	○	○	○	○
bei mittelschwerer Demenz	○	○	○	○	○	○	○	○
bei schwerer Demenz	○	○	○	○	○	○	○	○

14. Wie gut ist Ihrer Meinung nach die Wirkung von Donepezil auf die kognitiven Fähigkeiten Ihrer Patient:innen?

	stark negative Wirkung						stark positive Wirkung	kann ich nicht beurteilen
bei leichten kognitiven Einschränkungen	○	○	○	○	○	○	○	○
bei leichter Demenz	○	○	○	○	○	○	○	○
bei mittelschwerer Demenz	○	○	○	○	○	○	○	○
bei schwerer Demenz	○	○	○	○	○	○	○	○

15. Wie gut ist Ihrer Meinung nach die Wirkung von Donepezil auf die Lebensqualität und die Aktivitäten des täglichen Lebens Ihrer Patient:innen?

	stark negative Wirkung						stark positive Wirkung	kann ich nicht beurteilen
bei leichten kognitiven Einschränkungen	○	○	○	○	○	○	○	○
bei leichter Demenz	○	○	○	○	○	○	○	○
bei mittelschwerer Demenz	○	○	○	○	○	○	○	○
bei schwerer Demenz	○	○	○	○	○	○	○	○

16. Wie gut ist Ihrer Meinung nach die Wirkung von Donepezil auf die Verhaltensparameter Ihrer Patient:innen?

mit Verhaltensparametern sind in diesem Kontext Begleiterscheinungen, wie Depressionen, Wut, Ärger, Psychosen, etc. zu verstehen

	stark negative Wirkung						stark positive Wirkung	kann ich nicht beurteilen
bei leichten kognitiven Einschränkungen	○	○	○	○	○	○	○	○
bei leichter Demenz	○	○	○	○	○	○	○	○
bei mittelschwerer Demenz	○	○	○	○	○	○	○	○
bei schwerer Demenz	○	○	○	○	○	○	○	○

17. Wie gut ist Ihrer Meinung nach die Wirkung von Galantamin auf die kognitiven Fähigkeiten Ihrer Patient:innen?

	stark negative Wirkung						stark positive Wirkung	kann ich nicht beurteilen
bei leichten kognitiven Einschränkungen	○	○	○	○	○	○	○	○
bei leichter Demenz	○	○	○	○	○	○	○	○
bei mittelschwerer Demenz	○	○	○	○	○	○	○	○
bei schwerer Demenz	○	○	○	○	○	○	○	○

18. Wie gut ist Ihrer Meinung nach die Wirkung von Galantamin auf die Lebensqualität und die Aktivitäten des täglichen Lebens Ihrer Patient:innen?

	stark negative Wirkung						stark positive Wirkung	kann ich nicht beurteilen
bei leichten kognitiven Einschränkungen	○	○	○	○	○	○	○	○
bei leichter Demenz	○	○	○	○	○	○	○	○
bei mittelschwerer Demenz	○	○	○	○	○	○	○	○
bei schwerer Demenz	○	○	○	○	○	○	○	○

19. Wie gut ist Ihrer Meinung nach die Wirkung von Galantamin auf die Verhaltensparameter Ihrer Patient:innen?

mit Verhaltensparametern sind in diesem Kontext Begleiterscheinungen, wie Depressionen, Wut, Ärger, Psychosen, etc. zu verstehen

	stark negative Wirkung						stark positive Wirkung	kann ich nicht beurteilen
bei leichten kognitiven Einschränkungen	○	○	○	○	○	○	○	○
bei leichter Demenz	○	○	○	○	○	○	○	○
bei mittelschwerer Demenz	○	○	○	○	○	○	○	○
bei schwerer Demenz	○	○	○	○	○	○	○	○

20. Wie gut ist Ihrer Meinung nach die Wirkung von Antidepressiva auf die kognitiven Fähigkeiten Ihrer Patient:innen?

	stark negative Wirkung						stark positive Wirkung	kann ich nicht beurteilen
bei leichten kognitiven Einschränkungen	○	○	○	○	○	○	○	○
bei leichter Demenz	○	○	○	○	○	○	○	○
bei mittelschwerer Demenz	○	○	○	○	○	○	○	○
bei schwerer Demenz	○	○	○	○	○	○	○	○

21. Wie gut ist Ihrer Meinung nach die Wirkung von Antidepressiva auf die Lebensqualität und die Aktivitäten des täglichen Lebens Ihrer Patient:innen?

	stark negative Wirkung						stark positive Wirkung	kann ich nicht beurteilen
bei leichten kognitiven Einschränkungen	○	○	○	○	○	○	○	○
bei leichter Demenz	○	○	○	○	○	○	○	○
bei mittelschwerer Demenz	○	○	○	○	○	○	○	○
bei schwerer Demenz	○	○	○	○	○	○	○	○

22. Wie gut ist Ihrer Meinung nach die Wirkung von Antidepressiva auf die Verhaltensparameter Ihrer Patient:innen?

mit Verhaltensparametern sind in diesem Kontext Begleiterscheinungen, wie Depressionen, Wut, Ärger, Psychosen, etc. zu verstehen

	stark negative Wirkung						stark positive Wirkung	kann ich nicht beurteilen
bei leichten kognitiven Einschränkungen	○	○	○	○	○	○	○	○
bei leichter Demenz	○	○	○	○	○	○	○	○
bei mittelschwerer Demenz	○	○	○	○	○	○	○	○
bei schwerer Demenz	○	○	○	○	○	○	○	○

23. Wie gut ist Ihrer Meinung nach die Wirkung von Antipsychotika auf die kognitiven Fähigkeiten Ihrer Patient:innen?

	stark negative Wirkung						stark positive Wirkung	kann ich nicht beurteilen
bei leichten kognitiven Einschränkungen	○	○	○	○	○	○	○	○
bei leichter Demenz	○	○	○	○	○	○	○	○
bei mittelschwerer Demenz	○	○	○	○	○	○	○	○
bei schwerer Demenz	○	○	○	○	○	○	○	○

24. Wie gut ist Ihrer Meinung nach die Wirkung von Antipsychotika auf die Lebensqualität und die Aktivitäten des täglichen Lebens Ihrer Patient:innen?

	stark negative Wirkung						stark positive Wirkung	kann ich nicht beurteilen
bei leichten kognitiven Einschränkungen	○	○	○	○	○	○	○	○
bei leichter Demenz	○	○	○	○	○	○	○	○
bei mittelschwerer Demenz	○	○	○	○	○	○	○	○
bei schwerer Demenz	○	○	○	○	○	○	○	○

25. Wie gut ist Ihrer Meinung nach die Wirkung von Antipsychotika auf die Verhaltensparameter Ihrer Patient:innen?

mit Verhaltensparametern sind in diesem Kontext Begleiterscheinungen, wie Depressionen, Wut, Ärger, Psychosen, etc. zu verstehen

	stark negative Wirkung						stark positive Wirkung	kann ich nicht beurteilen
bei leichten kognitiven Einschränkungen	○	○	○	○	○	○	○	○
bei leichter Demenz	○	○	○	○	○	○	○	○
bei mittelschwerer Demenz	○	○	○	○	○	○	○	○
bei schwerer Demenz	○	○	○	○	○	○	○	○

---

26. Anhand welcher Parameter haben Sie die Wirksamkeit der medikamentösen Therapien auf die kognitiven Fähigkeiten festgemacht?

27. Anhand welcher Parameter haben Sie die Wirksamkeit der medikamentösen Therapien auf die Lebensqualität und die Aktivitäten des täglichen Lebens festgemacht?

28. Anhand welcher Parameter haben Sie die Wirksamkeit der medikamentösen Therapien auf das Verhalten festgemacht?

29. Bitte geben Sie die Häufigkeit an, in der Sie die folgenden Medikamente zur

Behandlung bei Demenz einsetzen

	sehr selten	selten	häufig	besonders häufig	nie
Rivastigmin	○	○	○	○	○
Memantin	○	○	○	○	○
Donepezil	○	○	○	○	○
Galantamin	○	○	○	○	○
Antidepressiva	○	○	○	○	○
Antipsychotika	○	○	○	○	○
Sonstiges häufig verschriebenes Medikament	○	○	○	○	○

30. Weshalb bevorzugen Sie den Gebrauch der Medikamente, welche Sie besonders häufig Verschrieben haben?

31. Welche der folgenden nicht-medikamentösen Verfahren zur Behandlung von Demenz kennen Sie?

- ☐ Ergotherapie
- ☐ Erinnerungstherapie
- ☐ körperliche Aktivität
- ☐ Verhaltenstherapie
- ☐ Kunst-/Musiktherapie
- ☐ kognitive Stimulationstherapie
- ☐ Sonstige ______

- ☐ keines der genannten Verfahren

32. Wie häufig empfehlen Sie, bei Patient:innen mit einer Demenzerkrankung, eine psychosoziale Intervention zur Behandlung von Demenz?

○	○	○	○	○	○
**nie**	**< 25%** der Patient:innen	**< 50%** der Patient:innen	**> 50%** der Patient:innen	**> 75%** der Patient:innen	**immer**

33. Aus welchen Gründen empfehlen Sie nicht-medikamentöse Interventionen?

Bitte geben Sie vor der Antwort, auch die betreffende Behandlungsform an.

______

______

34. Wie gut ist Ihrer Meinung nach die Wirkung von Ergotherapie auf die kognitiven Fähigkeiten Ihrer Patient:innen?

	stark negative Wirkung						starke positive Wirkung	kann ich nicht beurteilen
bei leichten kognitiven Einschränkungen	○	○	○	○	○	○	○	○
bei leichter Demenz	○	○	○	○	○	○	○	○
bei mittelschwerer Demenz	○	○	○	○	○	○	○	○
bei schwerer Demenz	○	○	○	○	○	○	○	○

35. Wie gut ist Ihrer Meinung nach die Wirkung von Ergotherapie auf die Lebensqualität und die Aktivitäten des täglichen Lebens Ihrer Patient:innen?

	stark negative Wirkung						stark positive Wirkung	kann ich nicht beurteilen
bei leichten kognitiven Einschränkungen	○	○	○	○	○	○	○	○
bei leichter Demenz	○	○	○	○	○	○	○	○
bei mittelschwerer Demenz	○	○	○	○	○	○	○	○
bei schwerer Demenz	○	○	○	○	○	○	○	○

36. Wie gut ist Ihrer Meinung nach die Wirkung von Ergotherapie auf die Verhaltensparameter Ihrer Patient:innen?

mit Verhaltensparametern sind in diesem Kontext Begleiterscheinungen, wie Depressionen, Wut, Ärger, Psychosen, etc. zu verstehen

	stark negative Wirkung						stark positive Wirkung	kann ich nicht beurteilen
bei leichten kognitiven Einschränkungen	○	○	○	○	○	○	○	○
bei leichter Demenz	○	○	○	○	○	○	○	○
bei mittelschwerer Demenz	○	○	○	○	○	○	○	○
bei schwerer Demenz	○	○	○	○	○	○	○	○

37. Wie gut ist Ihrer Meinung nach die Wirkung von Erinnerungstherapie auf die kognitiven Fähigkeiten Ihrer Patient:innen?

	stark negative Wirkung						stark positive Wirkung	kann ich nicht beurteilen
bei leichten kognitiven Einschränkungen	○	○	○	○	○	○	○	○
bei leichter Demenz	○	○	○	○	○	○	○	○
bei mittelschwerer Demenz	○	○	○	○	○	○	○	○
bei schwerer Demenz	○	○	○	○	○	○	○	○

38. Wie gut ist Ihrer Meinung nach die Wirkung von Erinnerungstherapie auf die Lebensqualität und die Aktivitäten des täglichen Lebens Ihrer Patient:innen?

	stark negative Wirkung						stark positive Wirkung	kann ich nicht beurteilen
bei leichten kognitiven Einschränkungen	○	○	○	○	○	○	○	○
bei leichter Demenz	○	○	○	○	○	○	○	○
bei mittelschwerer Demenz	○	○	○	○	○	○	○	○
bei schwerer Demenz	○	○	○	○	○	○	○	○

39. Wie gut ist Ihrer Meinung nach die Wirkung von Erinnerungstherapie auf die Verhaltensparameter Ihrer Patient:innen?

mit Verhaltensparametern sind in diesem Kontext Begleiterscheinungen, wie Depressionen, Wut, Ärger, Psychosen, etc. zu verstehen

	stark negative Wirkung						stark positive Wirkung	kann ich nicht beurteilen
bei leichten kognitiven Einschränkungen	○	○	○	○	○	○	○	○
bei leichter Demenz	○	○	○	○	○	○	○	○
bei mittelschwerer Demenz	○	○	○	○	○	○	○	○
bei schwerer Demenz	○	○	○	○	○	○	○	○

40. Wie gut ist Ihrer Meinung nach die Wirkung von körperlicher Aktivität auf die kognitiven Fähigkeiten Ihrer Patient:innen?

	stark negative Wirkung						stark positive Wirkung	kann ich nicht beurteilen
bei leichten kognitiven Einschränkungen	○	○	○	○	○	○	○	○
bei leichter Demenz	○	○	○	○	○	○	○	○
bei mittelschwerer Demenz	○	○	○	○	○	○	○	○
bei schwerer Demenz	○	○	○	○	○	○	○	○

41. Wie gut ist Ihrer Meinung nach die Wirkung von körperlicher Aktivität auf die Lebensqualität und die Aktivitäten des täglichen Lebens Ihrer Patient:innen?

	stark negative Wirkung						stark positive Wirkung	kann ich nicht beurteilen
bei leichten kognitiven Einschränkungen	○	○	○	○	○	○	○	○
bei leichter Demenz	○	○	○	○	○	○	○	○
bei mittelschwerer Demenz	○	○	○	○	○	○	○	○
bei schwerer Demenz	○	○	○	○	○	○	○	○

42. Wie gut ist Ihrer Meinung nach die Wirkung von körperlicher Aktivität auf die Verhaltensparameter Ihrer Patient:innen?

mit Verhaltensparametern sind in diesem Kontext Begleiterscheinungen, wie Depressionen, Wut, Ärger, Psychosen, etc. zu verstehen

	stark negative Wirkung						stark positive Wirkung	kann ich nicht beurteilen
bei leichten kognitiven Einschränkungen	○	○	○	○	○	○	○	○
bei leichter Demenz	○	○	○	○	○	○	○	○
bei mittelschwerer Demenz	○	○	○	○	○	○	○	○
bei schwerer Demenz	○	○	○	○	○	○	○	○

43. Wie gut ist Ihrer Meinung nach die Wirkung von Verhaltenstherapie auf die kognitiven Fähigkeiten Ihrer Patient:innen?

	stark negative Wirkung						stark positive Wirkung	kann ich nicht beurteilen
bei leichten kognitiven Einschränkungen	○	○	○	○	○	○	○	○
bei leichter Demenz	○	○	○	○	○	○	○	○
bei mittelschwerer Demenz	○	○	○	○	○	○	○	○
bei schwerer Demenz	○	○	○	○	○	○	○	○

44. Wie gut ist Ihrer Meinung nach die Wirkung von Verhaltenstherapie auf die Lebensqualität und die Aktivitäten des täglichen Lebens Ihrer Patient:innen?

	stark negative Wirkung						stark positive Wirkung	kann ich nicht beurteilen
bei leichten kognitiven Einschränkungen	○	○	○	○	○	○	○	○
bei leichter Demenz	○	○	○	○	○	○	○	○
bei mittelschwerer Demenz	○	○	○	○	○	○	○	○
bei schwerer Demenz	○	○	○	○	○	○	○	○

45. Wie gut ist Ihrer Meinung nach die Wirkung von Verhaltenstherapie auf die Verhaltensparameter Ihrer Patient:innen?

mit Verhaltensparametern sind in diesem Kontext Begleiterscheinungen, wie Depressionen, Wut, Ärger, Psychosen, etc. zu verstehen

	stark negative Wirkung						stark positive Wirkung	kann ich nicht beurteilen
bei leichten kognitiven Einschränkungen	○	○	○	○	○	○	○	○
bei leichter Demenz	○	○	○	○	○	○	○	○
bei mittelschwerer Demenz	○	○	○	○	○	○	○	○
bei schwerer Demenz	○	○	○	○	○	○	○	○

46. Wie gut ist Ihrer Meinung nach die Wirkung von Kunst-/Musiktherapie auf die kognitiven Fähigkeiten Ihrer Patient:innen?

	stark negative Wirkung						stark positive Wirkung	kann ich nicht beurteilen
bei leichten kognitiven Einschränkungen	○	○	○	○	○	○	○	○
bei leichter Demenz	○	○	○	○	○	○	○	○
bei mittelschwerer Demenz	○	○	○	○	○	○	○	○
bei schwerer Demenz	○	○	○	○	○	○	○	○

47. Wie gut ist Ihrer Meinung nach die Wirkung von Kunst-/Musiktherapie auf die Lebensqualität und die Aktivitäten des täglichen Lebens Ihrer Patient:innen?

	stark negative Wirkung						stark positive Wirkung	kann ich nicht beurteilen
bei leichten kognitiven Einschränkungen	○	○	○	○	○	○	○	○
bei leichter Demenz	○	○	○	○	○	○	○	○
bei mittelschwerer Demenz	○	○	○	○	○	○	○	○
bei schwerer Demenz	○	○	○	○	○	○	○	○

48. Wie gut ist Ihrer Meinung nach die Wirkung von Kunst-/Musiktherapie auf die Verhaltensparameter Ihrer Patient:innen?

mit Verhaltensparametern sind in diesem Kontext Begleiterscheinungen, wie Depressionen, Wut, Ärger, Psychosen, etc. zu verstehen

	stark negative Wirkung						stark positive Wirkung	kann ich nicht beurteilen
bei leichten kognitiven Einschränkungen	○	○	○	○	○	○	○	○
bei leichter Demenz	○	○	○	○	○	○	○	○
bei mittelschwerer Demenz	○	○	○	○	○	○	○	○
bei schwerer Demenz	○	○	○	○	○	○	○	○

49. Wie gut ist Ihrer Meinung nach die Wirkung von kognitiver Stimulationstherapie auf die kognitiven Fähigkeiten Ihrer Patient:innen?

	stark negative Wirkung						stark positive Wirkung	kann ich nicht beurteilen
bei leichten kognitiven Einschränkungen	○	○	○	○	○	○	○	○
bei leichter Demenz	○	○	○	○	○	○	○	○
bei mittelschwerer Demenz	○	○	○	○	○	○	○	○
bei schwerer Demenz	○	○	○	○	○	○	○	○

50. Wie gut ist Ihrer Meinung nach die Wirkung von kognitiver Stimulationstherapie auf die Lebensqualität und die Aktivitäten des täglichen Lebens Ihrer Patient:innen?

	stark negative Wirkung						stark positive Wirkung	kann ich nicht beurteilen
bei leichten kognitiven Einschränkungen	○	○	○	○	○	○	○	○
bei leichter Demenz	○	○	○	○	○	○	○	○
bei mittelschwerer Demenz	○	○	○	○	○	○	○	○
bei schwerer Demenz	○	○	○	○	○	○	○	○

51. Wie gut ist Ihrer Meinung nach die Wirkung von kognitiver Stimulationstherapie auf die Verhaltensparameter Ihrer Patient:innen?

mit Verhaltensparametern sind in diesem Kontext Begleiterscheinungen, wie Depressionen, Wut, Ärger, Psychosen, etc. zu verstehen

	stark negative Wirkung						stark positive Wirkung	kann ich nicht beurteilen
bei leichten kognitiven Einschränkungen	○	○	○	○	○	○	○	○
bei leichter Demenz	○	○	○	○	○	○	○	○
bei mittelschwerer Demenz	○	○	○	○	○	○	○	○
bei schwerer Demenz	○	○	○	○	○	○	○	○

Seite 20

52. Anhand welcher Parameter haben Sie die Wirksamkeit der nicht-medikamentösen Therapien auf die kognitiven Fähigkeiten festgemacht?

53. Anhand welcher Parameter haben Sie die Wirksamkeit der nicht-medikamentösen Therapien auf die Lebensqualität und die Aktivitäten des täglichen Lebens festgemacht?

54. Anhand welcher Parameter haben Sie die Wirksamkeit der nicht-medikamentösen Therapien auf das Verhalten festgemacht?

55. Welche Intervention hat Ihrer Meinung nach einen größeren positiven Effekt auf die kognitive Leistungsfähigkeit Ihrer Patient:innen?

- ○ medikamentöse Behandlung
- ○ psychosoziale Intervention
- ○ gleichermaßen

56. Welche Intervention hat Ihrer Meinung nach einen größeren positiven Effekt auf die Lebensqualität und die Aktivitäten des täglichen Lebens Ihrer Patient:innen?

- ○ medikamentöse Behandlung
- ○ psychosoziale Intervention
- ○ gleichermaßen

57. Welche Intervention hat Ihrer Meinung nach einen größeren positiven Effekt auf die Verhaltensparameter Ihrer Patient:innen?

- ○ medikamentöse Behandlung
- ○ psychosoziale Intervention
- ○ gleichermaße

58. Gibt es psychosoziale Interventionen, welche extrabudgetär verschrieben werden können?

- ○ Ja
- ○ Nein

59. Welche psychosozialen Interventionen können extrabudgetär verschrieben werden?

- [ ] Ergotherapie
- [ ] Erinnerungstherapie
- [ ] körperliche Aktivität
- [ ] Kunst-/Musiktherapie
- [ ] kognitive Stimulationstherapie
- [ ] Verhaltenstherapie
- [ ] Sonstige

60. Welche Barrieren erschweren, Ihrer Meinung nach, die Verschreibung bzw. Empfehlung psychosozialer Interventionen bei Demenz besonders stark?

61. Welche der folgenden Gründe betrachten Sie als Barriere für die Verschreibung psychosozialer Interventionen bei Demenz?

- ☐ Mangelnde Information über Angebote
- ☐ Mangelnde Evidenz
- ☐ Mangelnde Nachfrage von Patient:innen/Angehörigen
- ☐ Mangelnde Informationen über die Möglichkeiten der Verschreibung
- ☐ Mangelndes Vertrauen in Fähigkeiten der Therapeut:innen
- ☐ Skepsis der Patient:innen/Angehörigen gegenüber der Verfahren
- ☐ Regress-Ängste
- ☐ Fehlende Angebote in der Region
- ☐ Zu starke Einschränkungen Seitens der Patient:innen
- ☐ zu späte Demenz-Diagnose
- ☐ zu schlechte Prognose der Demenz-Erkrankung
- ☐ Mangel an Patient:innen mit Demenz-Diagnose

62. Haben Sie bereits multimodale* Therapieformen zur Behandlung von Demenz eingesetzt?

*mit multimodaler Therapie ist hier der kombinierte Einsatz unterschiedlicher Therapieformen, wie z.B die Kombination von pharmakologischen Interventionsmaßnahmen, mit psychosozialen Interventionen gemeint

- ○ Ja
- ○ Nein

63. Wie häufig verwenden Sie multimodale Therapien zur Behandlung von Demenz?

○ nie ○ sehr selten ○ selten ○ oft ○ sehr oft ○ immer

64. Wenn ja, weshalb haben Sie sich dazu entschieden, multimodale Therapien einzusetzen?

65. Wie könnte man die Aufklärung in Bezug auf die Behandlungsmöglichkeiten ihrer Meinung nach verbessern?

Infoseite

Wenn Sie mehr über nicht-medikamentöse Interventionen wissen möchten, können Sie gerne unsere Webseite besuchen:
mytuc.org/nwbx

Vielen Dank für Ihre Teilnahme!

Ich möchte mich ganz herzlich für Ihre Mithilfe bedanken.

Diese Studie dient dem Vergleich von medikamentöser und nicht-medikamentöser Therapie bei Menschen mit Demenz. Sollten während der Umfrage Fragen aufgekommen sein, können Sie mich gerne kontaktieren.

E-Mail: lea.pickard@gmx.de

Ihre Antworten wurden gespeichert, Sie können das Browser-Fenster nun schließen.
