# Supplementary material for: General practitioners’ and medical students’ current knowledge and attitudes toward non-pharmacological interventions for dementia
Source: Front Med (Lausanne). 2025 Jul 23;12:1573251. doi: 10.3389/fmed.2025.1573251 (PMC12325421; doi:10.3389/fmed.2025.1573251)
Supplement: Supplementary file 5 [file Data_Sheet_4.pdf]

Einzelinterview mit Allgemeinmediziner_innen

Sind Sie damit einverstanden, dass unser Gespräch aufgezeichnet wird?

Ort:
Datum:
Geschlecht des Teilnehmers/ der Teilnehmerin:

Welches ist Ihre genaue Berufsbezeichnung?
Welches ist Ihr Spezialgebiet?
In welchem Bundesland haben Sie studiert?
In welchem Bundesland arbeiten Sie?
Wie alt sind Sie?

Was assoziieren Sie mit Ergotherapie?
Welche Rolle spielte das Thema Ergotherapie in Ihrem Studium?
(War es Prüfungsbestandteil?)
Welche Erfahrungen haben Sie mit Ergotherapie?
Was halten Sie von dem ergotherapeutischen Ansatz (positive Aspekte, Bedenken)?
(Haben Sie Ergotherapie bereits selbst verschrieben?)
(Wie reagieren Patienten darauf, wenn Sie ihnen Ergotherapie empfehlen?)
((In welchen Fällen?))
((Auch bei Demenz?))

Was assoziieren Sie mit Verhaltenstherapie?
Welche Rolle spielte das Thema Verhaltenstherapie in Ihrem Studium?
(War es Prüfungsbestandteil?)
Welche Erfahrungen haben Sie mit Verhaltenstherapie?
Was halten Sie von dem verhaltenstherapeutischen Ansatz (positive Aspekte, Bedenken)?
(Haben Sie Verhaltenstherapie bereits selbst verschrieben?)
(Wie reagieren Patienten darauf, wenn Sie ihnen Verhaltenstherapie empfehlen?)
((In welchen Fällen?))
((Auch bei Demenz?))

Was könnte Sie dazu veranlassen, mehr Ergo-/ Verhaltenstherapie zu verschreiben?

Was veranlasst sie dazu, eine Demenz-Diagnose zu stellen?
Wie stellen Sie eine Demenz-Diagnose?
Sind Sie mit dem Verfahren zufrieden?

Welche Erfahrungen haben Sie mit Menschen mit Demenz gemacht?
Welche Wünsche und Bedenken äußern Menschen mit Demenz besonders häufig?
Welche Schwierigkeiten sehen Sie in der Versorgung von Menschen mit Demenz?

Welche Erfahrungen haben Sie mit Angehörigen von Menschen mit Demenz gemacht?
Welche Wünsche und Bedenken äußern Angehörige von Menschen mit Demenz häufig?
Welche Schwierigkeiten sehen Sie in der Versorgung von Angehörigen von Menschen mit Demenz?

Welche Therapieansätze/ Hilfsangebote halten Sie für besonders geeignet für Menschen mit Demenz?
(Welche noch?/ Noch welche?)
Welche Therapieansätze/ Hilfsangebote halten Sie für besonders geeignet für Angehörige von Menschen mit Demenz?
(Welche noch?/ Noch welche?)

Denken Sie, Allgemeinmediziner_innen sind ausreichend geschult in der Erkennung von Demenz?
Denken Sie, Allgemeinmediziner_innen sind ausreichend geschult für die Vermittlung passender Therapieansätze/ Hilfsangebote für die Menschen mit Demenz und ihre Angehörigen?
(Was halten Sie von einem Demenz-Abzeichen für Ärzt_innen?)

Empfinden Sie die Beschlüsse des gemeinsamen Bundesausschusses als Hilfe oder als Einschränkung für Ihren Arbeitsalltag/ Ihre Verschreibungspraxis?
(Inwiefern?)
(Inwieweit?)

Was halten Sie von einer Blankoverordnung (bei der ein_e Therapeut_in selbst entscheiden kann, welche Behandlung er/ sie durchführt)?

Wie würden Sie den Weg von einem Arztbesuch zu einer Intervention beschreiben?
(Wie, wenn die Intervention in der Häuslichkeit stattfindet?)

Wie erfahren Sie von neuen Interventionen?
(Wie kann man die Ärzteschaft am besten erreichen?)

Welche Informationen über den Patienten/ die Patientin und den Behandlungsfortgang benötigen Sie/ wünschen Sie sich?
(Wie kleinschrittig/ ausführlich?)
Auf welchem Wege möchten Sie diese Informationen erhalten?
(Wie häufig?)

Haben Sie weitere Hinweise, worauf bei der Konzeption einer interdisziplinären nicht-medikamentösen Intervention geachtet werden sollte (Abläufe, Vorgaben, ...)?

Das Interview ist fast beendet:
Haben Sie sich wohl gefühlt?
Haben Sie das Gefühl, wahrheitsgemäß geantwortet zu haben?

Vielen Dank.
